# Systematic discovery of pseudomonad genetic factors involved in sensitivity to tailocins

**DOI:** 10.1038/s41396-021-00921-1

**Published:** 2021-03-01

**Authors:** Sean Carim, Ashley L. Azadeh, Alexey E. Kazakov, Morgan N. Price, Peter J. Walian, Lauren M. Lui, Torben N. Nielsen, Romy Chakraborty, Adam M. Deutschbauer, Vivek K. Mutalik, Adam P. Arkin

**Affiliations:** 1 Department of Plant and Microbial Biology, University of California, Berkeley, CA USA; 2 Innovative Genomics Institute, University of California, Berkeley, CA USA; 3 Environmental Genomics and Systems Biology, Lawrence Berkeley National Laboratory, Berkeley, CA USA; 4 Molecular Biophysics and Integrated Bioimaging, Lawrence Berkeley National Laboratory, Berkeley, CA USA; 5 Climate and Ecosystem Sciences, Lawrence Berkeley National Laboratory, Berkeley, CA USA; 6 Department of Bioengineering, University of California, Berkeley, CA USA

**Keywords:** Functional genomics, Microbial ecology, Bacterial genetics, Soil microbiology

## Abstract

Tailocins are bactericidal protein complexes produced by a wide variety of bacteria that kill closely related strains and may play a role in microbial community structure. Thanks to their high specificity, tailocins have been proposed as precision antibacterial agents for therapeutic applications. Compared to tailed phages, with whom they share an evolutionary and morphological relationship, bacterially produced tailocins kill their host upon production but producing strains display resistance to self-intoxication. Though lipopolysaccharide (LPS) has been shown to act as a receptor for tailocins, the breadth of factors involved in tailocin sensitivity, and the mechanisms behind resistance to self-intoxication, remain unclear. Here, we employed genome-wide screens in four non-model pseudomonads to identify mutants with altered fitness in the presence of tailocins produced by closely related pseudomonads. Our mutant screens identified O-antigen composition and display as most important in defining sensitivity to our tailocins. In addition, the screens suggest LPS thinning as a mechanism by which resistant strains can become more sensitive to tailocins. We validate many of these novel findings, and extend these observations of tailocin sensitivity to 130 genome-sequenced pseudomonads. This work offers insights into tailocin–bacteria interactions, informing the potential use of tailocins in microbiome manipulation and antibacterial therapy.

## Introduction

Interference competition between closely related taxa is often mediated by the production and release of bacteriocins [1]. Bacteriocins are genetically encoded, ribosomally synthesized toxins that typically display a narrow killing spectrum [1]. The largest bacteriocins (2–10 MDa) are referred to as phage-tail-like bacteriocins or tailocins, and these are evolutionarily and morphologically related to bacteriophage tails, type VI secretion systems and extracellular contractile injection systems. They are encoded by single, contiguous biosynthetic gene clusters that resemble sequenced prophages [2], but lack capsid, integrase and terminase genes. Tailocins are either R-type or F-type, depending on whether they resemble *Myoviridae* or *Siphoviridae* phages, respectively.

In the laboratory, tailocin production can be induced by applying DNA damaging agents [2]. After tailocin particles are assembled in the cytoplasm, they are released by autolysis of the producing cell through activation of a dedicated lysis cassette. Tailocin target specificity is defined by its receptor-binding proteins (RBPs): tail fibers, tail spikes, and tail tips [3]. Recent studies have indicated that the RBPs are modular: they naturally undergo localized recombination [4], and they can be swapped in and out manually to engineer target specificity [5–8]. Following recognition and binding, tailocins kill target cells with very high potency, with one to a few particles sufficient for killing a sensitive cell [5, 9–11]. The mechanism of lethality is membrane depolarization, following insertion and puncture by the phage tail-derived structure [2]. In addition, there are indications that tailocin production can mediate changes in bacterial community structure and diversity [12–14]. Thus, they may be useful as a tool for targeted manipulation of microbiomes, with applications in both research and biotechnology.

Despite their importance and potential, our knowledge of cellular elements with which tailocins interact remains limited, an issue exacerbated by the scarcity of genetic tools for undomesticated bacterial isolates. Biochemical [15–17], and forward genetics studies [6, 7, 12, 18–20] in model strains suggest that tailocins bind to specific lipopolysaccharide (LPS) moieties, and that loss of these results in resistance. Nevertheless, it remains unclear whether LPS composition is the only determinant of tailocin sensitivity, and whether this differs between tailocin types. Meanwhile, tailocin-producing strains are resistant to their own tailocins (i.e., they avoid self-intoxication), but no systematic investigation for genetic factors responsible for this phenotype has yet been conducted. We aim to address the above knowledge gaps by leveraging resources developed previously by us and collaborators: (1) barcoded genome-wide transposon-insertion mutant libraries (RB-TnSeq) [21, 22] in environmental pseudomonads; and (2) a 130-member genome-sequenced pseudomonad isolate library.

In this work, we first investigate tailocin-mediated killing among a set of 12 *Pseudomonas* isolates. We identify the tailocin biosynthetic clusters in these strains, then induce and partially purify their tailocins. We characterize a subset of these tailocin samples via proteomics and transmission electron microscopy (TEM), then use them to screen RB-TnSeq [21] mutant libraries for differential tailocin sensitivity phenotypes in six separate tailocin–strain interactions. Our fitness assays: (1) establish O-specific antigen (OSA) composition and display as major determinants of tailocin sensitivity, and (2) show that disruptions to phospholipid (PL) retrograde and LPS anterograde transport weaken resistance to tailocin self-intoxication. Then, we examine the relationship between OSA biosynthetic genes and tailocin sensitivity at a larger scale, profiling sensitivity of our characterized tailocins across 130 genome-sequenced pseudomonads. We find that strains with the same overall OSA cluster typically display the same tailocin sensitivity pattern. However, OSA gene content alone is unable to explain all observed variance in sensitivity. Overall, this work represents the first systematic effort to identify genetic factors involved in sensitivity to tailocins. Our findings offer a more comprehensive and nuanced view on how bacteria can alter their sensitivity to tailocin-mediated interference.

## Results

### Identification, induction, and partial purification of tailocins

**Fig. 1:**
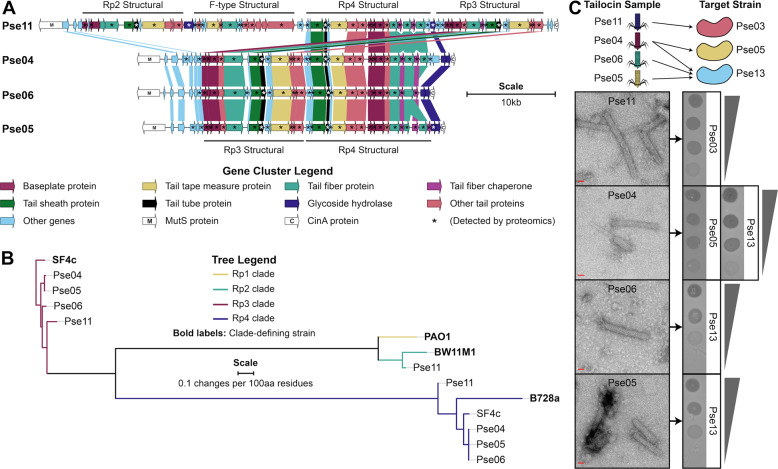
Characterization of select tailocin samples. Tailocins produced by our isolates Pse11, Pse04, Pse06, and Pse05 were characterized further in this study. **A** Tailocin biosynthetic gene clusters. Genes are colored by the presence of key words in their PHASTER [70] predicted annotations. Genes in the same group of orthologs (“orthogroup,” see Methods, Supplementary Table S3) are joined by a block of color. The products of starred (*) genes are observed in our partially purified tailocin samples using MudPIT proteomics (Methods, Supplementary Table S4). Additional data on these clusters can be found in Supplementary Table S2. **B** Phylogeny of R-type tailocins by tail tube protein sequence. These are delineated by clade as per their relationship to the following tailocins: *P. aeruginosa* PAO1 R2 pyocin (Rp1), *P. putida* BW11M1 R-type tailocin (Rp2), *P. fluorescens* SF4c R-type tailocin 1 (Rp3), and *P. syringae* B728a R-type tailocin (Rp4) [26]. **C** A schematic of tailocin–host interactions characterized in this work is presented (top). Characterization of five lethal tailocin interactions by TEM and by spotting fivefold dilution series of samples (bottom). Scale bar: 20 nm. Gray triangles illustrate the relative concentration of tailocins in the dilution series. Zones of inhibition formed by diluted samples are indicative of nonreplicative toxins, not phages. Spotting was done in triplicate and replicates gave identical data.

We first examined tailocin production and sensitivity in 12 *Pseudomonas* strains isolated from groundwater. These strains are classified into ten species under the genus “Pseudomonas_E” according to the Genome Taxonomy Database (GTDB) [23] (see Supplementary Table S1 for all strain information). To aid discussion, we will describe these 12 strains using codenames (“Pse##”) defined in Supplementary Table S1. We computationally searched for tailocin biosynthetic clusters in the 12 strains and found them in all except Pse13 (Methods, see Supplementary Table  S2 for a list of genes identified). All clusters were inserted only between the genes *mutS* and *cinA*, a known hotspot for prophage integration [24, 25] (Supplementary Fig. S1). These tailocin biosynthetic clusters vary in size (13.4–57.0 kb) and appear to encode 1–4 tailocin particles of both R- and F-type (Supplementary Fig. S1, Fig. 1A, and Supplementary Table S3). The R-type particles can be classified into subtypes Rp2, Rp3, or Rp4 based on their evolutionary relationships to different *Myoviridae* phages (Fig. 1B) [26]. Only Pse11 also encodes an F-type particle, and it resembles both the F2 pyocin of *P. aeruginosa* PAO1 [27] and the tail portion of *P. protegens* Pf-5 lambdoid prophage 06 [28].

To assess whether these gene clusters are functional and responsible for encoding tailocins, we employed published protocols to induce and produce tailocins from our 12 strains and evaluate their targeting spectra [25]. Briefly, we induced tailocin production in each strain by applying mitomycin C to mid-log cultures and collected all tailocins from the culture supernatant through ammonium sulfate precipitation (Methods). As a control, we also subjected mitomycin C untreated culture supernatant to the same precipitation protocols. We then assessed the targeting spectra of these tailocin samples by spotting each sample on each of the 12 strains for a 12 × 12 killing matrix (Supplementary Fig. S2). Consistent with the narrow target specificity expected of tailocins, lethality was sparse (15 killing interactions out of 144). Furthermore, no tailocin sample was toxic to its producing strain.

### Characterization of tailocin particles

We further characterized four of the tailocin samples (produced by Pse11, Pse04, Pse06, and Pse05). To examine the killing activity of our tailocins at lower concentrations, we spotted serial dilutions of the samples on sensitive strains. Spots made by the more diluted samples displayed reduced bacterial killing activity across the entire area of the zone of inhibition, and showed no distinct plaques (Fig. 1C). As reported earlier, this pattern is more typical of a diluted toxin, and not of a replicable killing agent like a phage [25]. To assess the protein composition of the tailocins and link them to structural genes, we subjected the samples to MudPIT LC/MS/MS proteomics analysis. Our proteomics data confirmed the presence of proteins for all possible tailocin particles encoded by our strains (Fig. 1A and Supplementary Table S4). Thus, each sample likely consists of a mixture of two or four particles (Fig. 1A). Our proteomics data do not indicate the presence of proteinaceous toxins, such as S-bacteriocins. Finally, we imaged each tailocin sample using TEM (Fig. 1C). Our micrographs showed an abundance of 20–25-nm wide, straight or slightly curved rods, resembling past microscopy reports of *Pseudomonas* R-tailocins [9, 16, 29]. Most particles were uncontracted, with lengths between approximately 110 and 170 nm. A few particles were contracted (see Fig. 1C Pse04 micrograph), with the 20–25-nm wide segment (likely the tail sheath) visibly shortened, and with a narrower ~18-nm segment (likely the tail tube) protruding from one side.

To discern which tailocin particles are responsible for our observed killing activity, we aimed to delete the putative baseplate genes (Fig. 1A) for each of the R-type particles encoded by the four producing strains. Single baseplate locus deletion is a well-established method for eliminating assembly of a single tailocin particle while preserving the assembly of other particles produced by the strain [30, 31]. We succeeded in generating seven of the nine possible baseplate mutants, partially purified tailocin samples from each mutant, and spotted them on sensitive strains (Methods, Supplementary Fig. S3). Killing activity was assigned to a particle by identifying which baseplate mutation eliminates killing achieved by the wildtype. We found that Pse05 Rp4 kills Pse13, Pse04 Rp3 kills Pse05, and Pse04 Rp4 kills Pse13. Deleting Pse06’s Rp3 particle, or, separately, its Rp4 particle, did not eliminate its ability to produce a killing agent against Pse13. In addition, we observe that killing of Pse03 by Pse11 is not mediated by the latter’s Rp4 particle, but are unable to specify which of its remaining particles are responsible (Supplementary Fig. S3). In the case of Pse04, we corroborate findings that encoding multiple tailocin particles increases the range of susceptible targets [30].

### Genome-wide mutant fitness assays identify gene functions involved in tailocin sensitivity

**Fig. 2:**
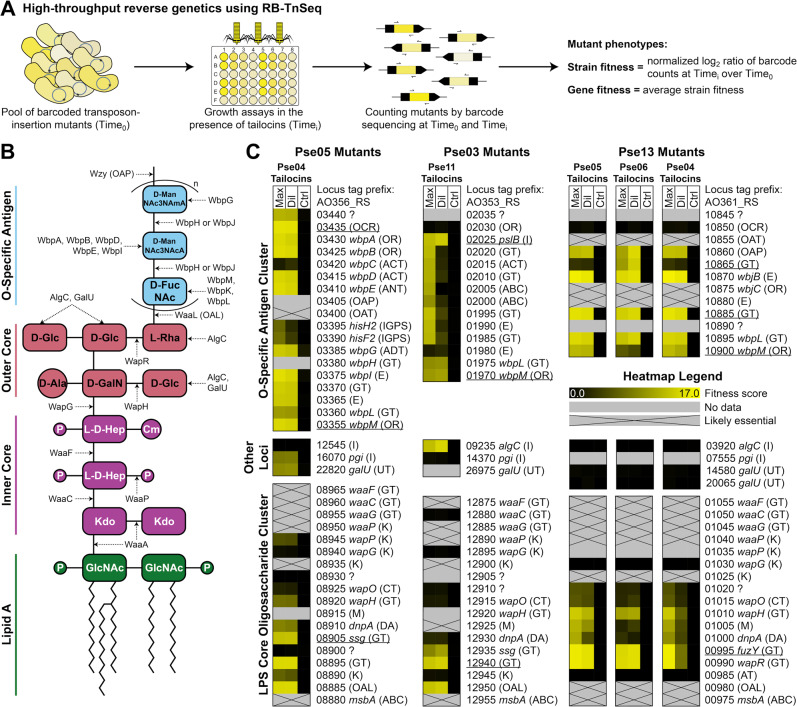
Genes implicated in tailocin sensitivity. **A** The RB-TnSeq approach for measuring normalized gene fitness. **B** Structure of the O-specific antigen LPS of *P. aeruginosa* PAO1 and enzymes involved in its assembly, based on King et al. [34]. Chemical moieties are abbreviated with the following definitions: Cm O-carbamoyl, D-Ala D-alanine, D-FucNAc 2-acetamido-2-deoxy-D-fucose, D-GalN D-galactosamine, D-Glc D-glucose, D-ManNAc3NAcA 2,3-diacetamido-2,3-dideoxy-D-mannuronate, D-ManNAc3NAmA 2-acetamido-3-acetamidino-2,3-dideoxy-D-mannuronate, GlcNAc N-acetyl-D-glucosamine, Kdo ketodeoxyoctonoate, L-D-Hep L-glycerol-D-manno-heptose, L-Rha L-rhamnose, P phosphorylation (with either one or multiple phosphates or phosphoethanolamines). Select gene function abbreviations are also included: OAL O-antigen ligase, OAP O-antigen polymerase. **C** Heatmaps depicting fitness scores for all genes in the LPS core oligosaccharide and O-specific antigen clusters, and those in other loci that confer tailocin resistance when mutated. Tailocins were supplied at two different concentrations: maximum concentration (“Max”) and a tenfold dilution from maximum (“Dil”). Tailocin-free buffer was supplied for control experiments (“Ctrl”). All fitness assays were performed in duplicate, and the average fitness is displayed. A gene lacking data means we did not obtain a transposon insertion in it [22]. Genes were annotated in two levels of detail depending on their homology to well-characterized genes. First, all genes were labeled with a function abbreviation. ABC ABC transporter subunit, ACT acetyltransferase, ADT amidotransferase, ANT aminotransferase, AT acyltransferase, C carbamoyltransferase, DA deacetylase, E epimerase, GT glycosyltransferase, I isomerase, IGPS imidazole glycerol phosphate synthase subunit, K kinase, M Mig-14-like, OAL O-antigen ligase, OAP O-antigen polymerase, OAT O-antigen translocase, OCR O-antigen chain length regulator, OR oxidoreductase, UT uridylyltransferase, ? unknown. Then, if a gene’s translation shared ≥60% identity with ≥90% coverage to a characterized gene in *P. aeruginosa* PAO1, *P. aeruginosa* PA103, or *P. fluorescens* SBW25, it was assigned the same gene name. See Supplementary Tables S6–S14 for read count, *t*-like statistic, and additional fitness data for these genes in Pse05, Pse03, and Pse13. Phenotypes of underlined genes have been validated by spotting tailocin samples on individual mutants (Supplementary Fig. S4 and Supplementary Table S15).

To study genes important in tailocin sensitivity, we sourced three pooled, barcoded, genome-wide transposon-insertion (RB-TnSeq) mutant libraries (for strains Pse05, Pse03, and Pse13) reported previously [22] (Supplementary Table S5). We then performed fitness experiments on these libraries, assaying five library and tailocin sample combinations (see Figs. 1C and 2A). In these experiments, selection pressure from the tailocins increases the relative abundance of resistant mutants. Changes in the relative abundance of mutants are monitored using next-generation sequencing via the BarSeq approach [21] to track DNA barcodes before and after tailocin treatment. These barcode sequencing data are calculated into fitness scores for each strain and each gene as described earlier [21] (Fig. 2A, Methods). Here, the positive gene fitness scores indicate genes conferring relative tailocin resistance when disrupted. Conversely, negative fitness scores indicate genes that confer relative tailocin sensitivity when disrupted.

Treatment with an antagonizing tailocin results in a few very high, replicable, positive gene fitness scores (up to +16), indicative of reliable selection for a very small subset of mutants (Fig. 2C). These genes are clearly important for tailocin sensitivity. However, the competitive advantage of the high fitness mutants in our pooled assays means mutants with weaker positive fitness have likely been severely outcompeted and eliminated from the population. Our earlier work with phage fitness assay experiments [32, 33], which, like here, involve the application of a stringent selective agent, suggests we focus our discussion on gene disruptions that confer the top positive fitness phenotypes in the presence of tailocins (see Methods for criteria). Genes that passed our criteria were few in number (44 total genes in three strains across five fitness assays) (detailed data on read count, *t*-like statistic, and additional fitness data for these genes is given in Supplementary Tables S6–S14 and experimental validations are given in Supplementary Fig. S4 and Supplementary Table S15). All but three of these genes lie within either the LPS core oligosaccharide biosynthetic gene cluster or the OSA biosynthetic gene cluster. The three remaining genes (orthologs of *pgi,* *galU,* and *algC*), while located elsewhere in the genome, are involved in the assembly of LPS monomers [34]. Notably, no gene disruptions in our strains’ common polysaccharide antigen (CPA) clusters (Pse05 AO356_RS11820-80; Pse03 AO353_RS10215-75; Pse13 AO361_RS11625-90), which are responsible for assembling a separate O-polysaccharide molecule [34], gave high enough fitness scores to pass our criteria.

While there is considerable heterogeneity in LPS gene content across Pse05, Pse03, and Pse13, we can still make some inferences about their functions using homology to well-characterized PAO1 LPS genes (Fig. 2B). The LPS core gene clusters in our strains are generally homologous to PAO1 and to each other (Supplementary Fig. S5 and Supplementary Table S16), so those genes are mostly assigned high confidence annotations as a result (see Fig. 2C caption). Variation in the LPS core clusters includes the absence of *wapP* in Pse03 and the weak homology of the *wapR* orthologs in Pse05 and Pse03. Meanwhile, only Pse05 has an OSA gene cluster homologous to PAO1 (Supplementary Fig. S6 and Supplementary Table S17), making that cluster the best annotated of its type on Fig. 2C.

### The O-specific antigen as the key factor in tailocin sensitivity

In Fig. 2C, we depict fitness score data for all genes in the LPS core and OSA clusters, and homologs of *algC*, *pgi*, and *galU*. Thus, Fig. 2C allows comparison of LPS biosynthetic genes by their importance in tailocin-mediated killing. Overall, the high fitness genes encode proteins that build LPS core monomers (*galU*, *algC*), assemble the LPS core (*wapH*, *wapR*), build OSA monomers (*wbpA*, *wbpB*, *wbpD*, *wbpE*, *wbpG*, *wbpI*, *wbpL*, *wbpM*, *pgi*, *pslB*, *algC*, *wbjB*), polymerize OSAs, regulate the length of OSAs, translocate OSAs, and ligate OSAs to the LPS core. Loss of any of these functions in *P. aeruginosa* is known to result in truncation or complete absence of one or both of the OSA and CPA [34], or, in the case of the chain length regulator, display of OSAs with altered length [34]. This suggests that, in our strains, intact, correct length OSA is necessary for tailocin-mediated killing, likely as a receptor for tailocin binding. This concurs with, and expands on, recent work with Rp4 tailocins in *P. syringae* [20, 35].

Since there are high fitness disruptions in orthologs of genes involved directly in LPS outer core assembly (*galU*, *wapH*, *wapR*), one could argue that some LPS outer core residues also serve as tailocin receptors. While there is evidence that other *Pseudomonas* tailocins target the outer core [12, 17, 36–38], the strong benefits of OSA mutations imply that OSA is the primary receptor for our studied tailocins. The phenotypes of (*galU*, *wapH*, *wapR*) disruption could reflect the loss of OSA, as the structures they assemble are required for OSA attachment [34]. Thus we only make claims about the role of the OSA in this work. For discussion of the role of LPS inner core biosynthetic enzymes, see Supplementary Note 1.

Phages are known to use both LPS moieties and membrane proteins as receptors [39], but tailocins are only known to use LPS moieties as receptors [12, 15, 17, 19, 20, 26, 35, 36]. Our data, which represents the most comprehensive genetic analysis so far of possible tailocin receptors, maintains this paradigm. In work recently published by ours and other groups [32], RB-TnSeq and a similar fitness assay design is used to find genes required for sensitivity to *Escherichia coli*, *Phaeobacter inhibens*, and *Salmonella enterica* phages [32, 33, 40], and the data recover their known membrane protein receptors. Thus, our methods employed here are capable of identifying non-essential membrane protein receptors, and the tailocins we tested do not depend on such factors.

### Disruption of specific OSA biosynthesis genes confers resistance to a subset of antagonistic tailocins

Challenging the Pse13 RB-TnSeq library with three different antagonistic tailocin samples allowed us to identify genes that are involved in sensitivity to some, but not all, of these tailocins. We identified two such genes, AO361_RS10865 and AO361_RS10900, both in the OSA biosynthetic cluster (Fig. 2C). Disruption of AO361_RS10865, which is a putative glycosyltransferase, confers greatly increased resistance to Pse06 tailocins, but has a small effect on resistance to Pse05 and Pse04 tailocins. In contrast, disruption of AO361_RS10900, an ortholog of WbpM (UDP-4,6-GlcNAc dehydratase) in *P. aeruginosa* PAO1 (78% identity, 100% coverage), confers resistance to Pse05 and Pse04 tailocins, but has a reduced effect on killing by Pse06 tailocins (Fig. 2C). We validated these phenotypes by spot tests on individual Pse13 mutants (Supplementary Fig. S4 and Supplementary Table S15). See Supplementary Note 2 for further review of the putative functions of AO361_RS10865 and AO361_RS10900. Our findings highlight the speed and fine resolution of RB-TnSeq fitness assays in identifying genes involved in tailocin resistance in non-model bacteria.

### Disruption of genes encoding outer membrane lipid asymmetry and LPS transport functions increases sensitivity to tailocin self-intoxication

**Fig. 3:**
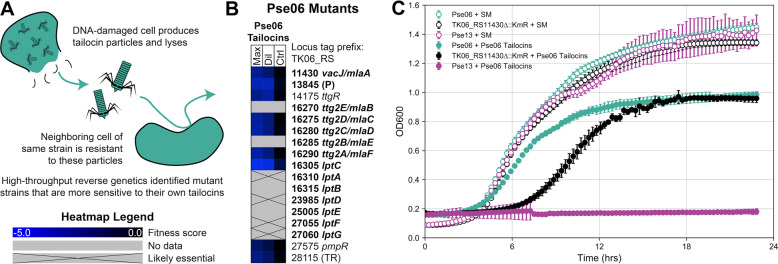
Genes implicated in resistance to tailocin self-intoxication. **A** Illustration of resistance to tailocin self-intoxication. **B** Heatmap depicting fitness data for transposon-insertion mutations in Pse06 that confer negative fitness when challenged by Pse06 tailocins. Tailocins were supplied at two different concentrations: maximum concentration (“Max”) and a tenfold dilution from maximum (“Dil”). In addition, tailocin-free buffer was supplied for control experiments (“Ctrl”). A gene lacking data means we did not obtain a transposon insertion in it [22]. Genes were annotated with gene names from either *P. aeruginosa* PAO1 or *P. putida* KT2440 if their amino acid sequence shared ≥60% identity with ≥90% coverage. *lptC* was additionally labeled thanks to its genomic context. Remaining genes were annotated with a function prediction: P peptidase, TR transcriptional repressor. Bold genes are putatively involved in maintaining LPS display at the outer leaflet of the outer membrane. See Supplementary Tables S18–S20 for read counts, *t*-like statistics, and additional fitness data for these genes. **C** Phenotypic validation by growth curve for the independently generated TK06_RS11430 (*vacJ/mlaA*) deletion in Pse06. SM SM buffer. The mutant displays increased sensitivity to Pse06 tailocins compared to Pse06 wild-type. Pse13 growth curves are included as a tailocin-sensitive control. These experiments were repeated (triplicate) and averages are plotted. Error bars: standard deviation.

A wide body of evidence shows that tailocin-producing strains are resistant to the tailocins they produce [4, 12], in a phenotype referred to as resistance to self-intoxication (Fig. 3A and Supplementary Fig. S2). Tailocins are believed to have evolved from prophages, so resistance to self-intoxication may have arisen from natural selection away from self-targeting, and toward targeting competing bacteria. Mechanistically, this is thought to occur through evolution of the tailocin’s RBPs [2]. Past work has shown that mutation of LPS biosynthetic genes resulting in loss of the OSA increases sensitivity to tailocin self-intoxication in *P. aeruginosa* [12, 37, 38]. Unlike with S-type bacteriocins, which are genetically transferred along with a cognate immunity gene, there are no known immunity genes for tailocins.

To investigate additional genes involved in resistance to tailocin self-intoxication, we challenged the Pse06 RB-TnSeq library with tailocins produced by wild-type Pse06, and looked for strong negative fitness (≤−2.0) effects. That is, we looked for mutants that show increased sensitivity when treated with tailocins as compared to the no-treatment control. These criteria yielded nine hits, depicted in Fig. 3B (detailed data on read count, *t*-like statistic, and additional fitness data for these genes are given in Supplementary Tables S18–S20, respectively). Six of the nine hits appear to have functions relevant to maintaining a sufficient density of LPS at the outer leaflet of the outer membrane (OM) (see bold genes in Fig. 3B) [41, 42]. Among these, four of the hits (TK06_RS11430, TK06_RS16275, TK06_RS16280, TK06_RS16290) are in the *mla* (maintenance of lipid asymmetry) pathway, which constitute a retrograde PL transport system involved in OM lipid homeostasis. The *mla* pathway removes PLs that mislocate to the outer leaflet of the OM so that dense packing of LPS, and OM integrity, is maintained [43]. Another hit is an ortholog of *lptC* (TK06_RS16305), encoding a component of the *lpt* LPS transport system that delivers LPS to the outer leaflet of the OM [44]. One more hit is a putative metalloprotease (TK06_RS13845) with weak homology (36% identity, 95% coverage) to *E. coli*’s BepA, a periplasmic chaperone or protease for OM proteins. BepA assists in the formation of LptD, an OM beta-barrel protein, and also degrades misassembled LptD [45]. Finally, the remaining three sensitivity hits—TK06_RS14175 (repressor of multidrug efflux pump [46]), TK06_RS27575 (quorum sensing response regulator [47]), and TK06_RS28115 (TetR family transcriptional regulator)—do not have discernible functions in LPS display. None of our nine hits localize to the tailocin biosynthetic cluster. This suggests that tailocins do not possess co-located immunity factors, which is common for other proteinaceous toxins (e.g., S-type bacteriocins, lactic acid bacteria type I and II bacteriocins, and type IV and VI secretion system effectors) [1, 48–51].

To validate these increased tailocin sensitivity phenotypes, the nine inferred sensitizing mutations were independently generated (see Methods), and the tailocin sensitivity phenotypes of these mutants were validated by measuring growth curves of planktonic cultures in the presence and absence of Pse06 tailocins. An exemplar set of growth curves for the TK06_RS11430 mutant is illustrated in Fig. 3C. Additional growth curves for the remaining eight disruptions are presented in Supplementary Fig. S7. In each case, these mutants exhibited impaired growth relative to wild-type Pse06 in the presence of the Pse06 tailocins. However, the mutants do not become completely sensitive to Pse06 tailocins, such as Pse13, a strain included as a sensitive control.

### The relationship between OSA biosynthetic gene content and tailocin sensitivity across an isolate library

**Fig. 4:**
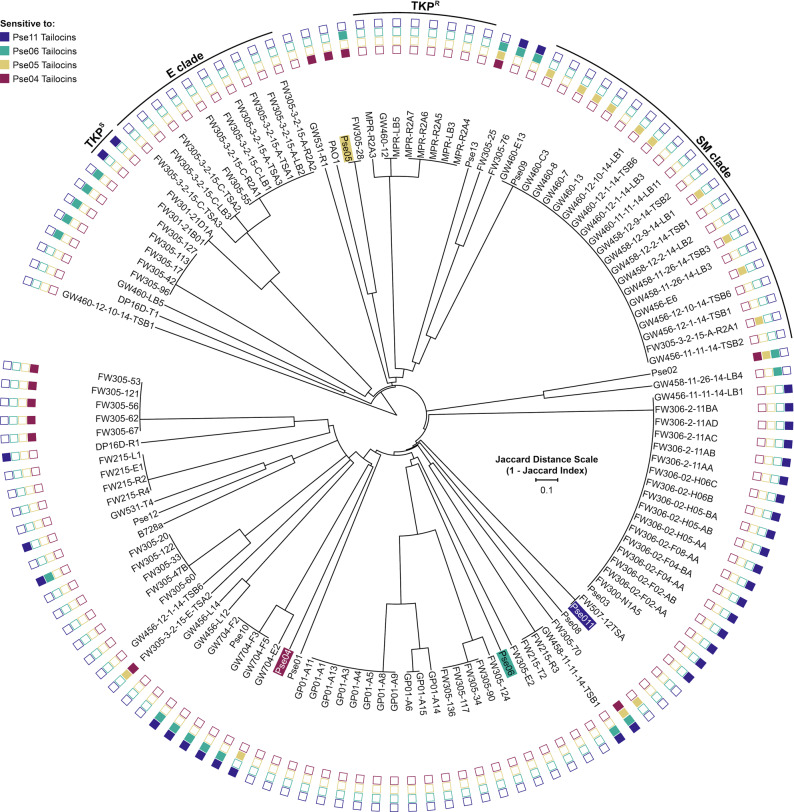
Hierarchical clustering of target strains by O-specific antigen biosynthetic cluster similarity, overlaid with tailocin sensitivity data. In all, 130 *Pseudomonas* strains were clustered by the similarity of their OSA clusters, assessed as Jaccard distance of orthogroups (OrthoFinder v2.2.7 [71]) found in the OSA cluster genes. See Methods for details on our approach to delineating OSA clusters. Tailocin-producing strain labels are highlighted in color. Shaded boxes at the outer edge of the tree indicate sensitivity of that strain to the correspondingly colored tailocin. To aid discussion of three clades of strains with interesting features, we named those clades after the most closely related species by 16S rRNA similarity: E clade (*P. extremorientalis*); TKP clade (*P*. sp. TKP); SM clade (*P. silesiensis*/*P.mandelii*). In this clustering, TKP strains are separated into two groups: TKP^S^ (Pse11 Tailocins sensitive) and TKP^R^ (resistant). For a phylogenetic tree of these strains, see Supplementary Fig. S8. For a comparative illustration of the OSA clusters encoded by these strains, see Supplementary Fig. S9 (for a full list of orthogroups, see Supplementary Table S22). For the killing matrix in table format, see Supplementary Table S6.

Since our mutant fitness data points to OSA composition and display as the key factors in tailocin sensitivity, we decided to examine the relationship between a target strain’s OSA biosynthetic gene cluster and its tailocin sensitivity among natural strain variants. To do this, we expanded sensitivity phenotyping of our four tailocin samples (Fig. 1C and Supplementary Table S21) to our in-house collection of 128 genome-sequenced groundwater *Pseudomonas* isolates (classified into 29 GTDB species under the genus “Pseudomonas_E” [23], Supplementary Table S1). We also included the well-studied human pathogen *P. aeruginosa* PAO1 and plant pathogen *P. syringae* B728a in our sensitivity assays. All 130 strains were hierarchically clustered by their OSA gene content similarity (see for a full list of orthogroups Supplementary Table S22, Methods). Figure  4 shows the tailocin sensitivity data decorated onto the OSA clustering of the 130 strains. Separately, we arranged the strains onto a phylogenetic tree built from a concatenation of 88 single-copy marker gene sequences to compare phylogenetic distance with tailocin sensitivity (Supplementary Fig. S8, Methods).

Generally, strains with similar or identical OSA clusters had the same tailocin sensitivity phenotypes. This can be seen across Fig. 4 or Supplementary Fig. S9 as members of the same dendrogram branch sharing the same pattern of sensitivity to the four tailocin samples. An exception to this is the “SM” clade, whose 21 members shared identical (to the nucleotide level) OSA genes (Supplementary Fig. S9) and a close phylogenetic relationship (>99.9% pairwise average nucleotide identity [ANI]) but vary in their sensitivity to Pse05 tailocins (11 sensitive, 10 resistant). We compared these strains at their *mla* and *lpt* loci and found that those, too, are identical. Finally, we performed a genome-wide association study (GWAS) on the SM strains using DBGWAS [52] that failed to find any genomic factors associated with Pse05 tailocin sensitivity with a *q*-value below 0.64. At this point, the genotypes responsible for the differential tailocin sensitivity in the SM clade are unknown to us, and additional work is needed to uncover them.

In addition, strains that cluster by OSA similarity are also typically similar across most of the genome (Fig. 4 and Supplementary Fig. S8), though again there are exceptions. The most notable exception here is among members of the “TKP” clade, all of whom group together phylogenetically (Supplementary Fig. S8). Two of the ten TKP strains (TKP^S^) display sensitivity to Pse11 tailocins by spot test, while the remaining eight strains (TKP^R^) are resistant. Comparison of the OSA clusters between TKP^S^ and TKP^R^ strains shows that they are highly divergent (Supplementary Fig. S9). Curiously, the OSA clusters of TKP^S^ strains closely resemble those encoded by strains in the “E” clade (which is resistant to Pse11 tailocins), but differ in one region that putatively encodes two hypothetical proteins and a sugar-modifying enzyme (Supplementary Fig. S9). Since loss of a single OSA biosynthetic gene can alter the tailocin sensitivity of a strain (Fig. 2C), we hypothesize that the genes in this variable region are responsible for the differential sensitivity of TKP strains to Pse11 tailocins. The opposite scenario is exemplified by Pse05 and PAO1, which are phylogenetically distinct (79.95% ANI), yet share similar OSA clusters and the same tailocin sensitivity pattern (Fig. 4).

Our comparative genomics analysis is in incomplete agreement with our fitness data, which emphasizes the importance of OSA on tailocin sensitivity. A loose relationship between OSA and sensitivity can be seen: strains with the same overall OSA cluster tend to have the same sensitivity. However, we also observe that variation in sensitivity can be achieved without apparent change in OSA gene sequence, exemplified by the SM strains. Thus, factors involved in tailocin sensitivity are more complex and nuanced than what we have been able to uncover here, and future experiments must be designed with this in mind.

## Discussion

In this work, we first systematically analyze the tailocin-encoding capabilities of *Pseudomonas* isolates, then partially purify and characterize the encoded tailocins. In the process, we use targeted mutagenesis to identify the specific tailocin particle responsible for three of five lethal tailocin–strain interactions. We confirmed that the Pse05 Rp4 tailocin particle kills Pse13, the Pse04 Rp3 tailocin particle kills Pse05, and the Pse04 Rp4 tailocin particle kills Pse13. For the remaining two interactions (Pse11 killing Pse03, Pse06 killing Pse13), the lack of genetic tools for these environmental isolates hampered our investigation. Nevertheless, a combination of our tailocin purification method, TEM images (Fig. 1C), spot testing of dilutions (Fig. 1C), proteomics data (Supplementary Table S4), and mutant fitness spectra (Fig. 2) strongly indicates that the killing activity by Pse11 and Pse06 is a feature of tailocins and not by other smaller toxic proteins.

We use our RB-TnSeq technology to uncover genetic determinants of tailocin sensitivity in the above-mentioned five lethal tailocin–strain interactions, and in an additional resistant tailocin–strain interaction (Figs. 2 and 3). Our results highlight the role of OSA composition and display as the key factors defining tailocin sensitivity, and do so at gene-level resolution. We identify individual OSA biosynthetic genes whose functions are involved in sensitivity to different specific tailocins. Subsequently, we assemble evidence supporting the role of LPS density in tailocin resistance, showing that mutations to retrograde PL transport and anterograde LPS transport systems increase the sensitivity of a strain to its own tailocins. Finally, we investigate the relationship between OSA gene content and tailocin sensitivity across 130 *Pseudomonas* strains. Strains with highly similar OSA clusters broadly share the same tailocin sensitivity pattern, even if they are phylogenetically distant, but exceptions exist. Our analysis suggests that genetic factors beyond those studied here are involved in tailocin sensitivity, warranting more nuanced investigation.

**Fig. 5:**
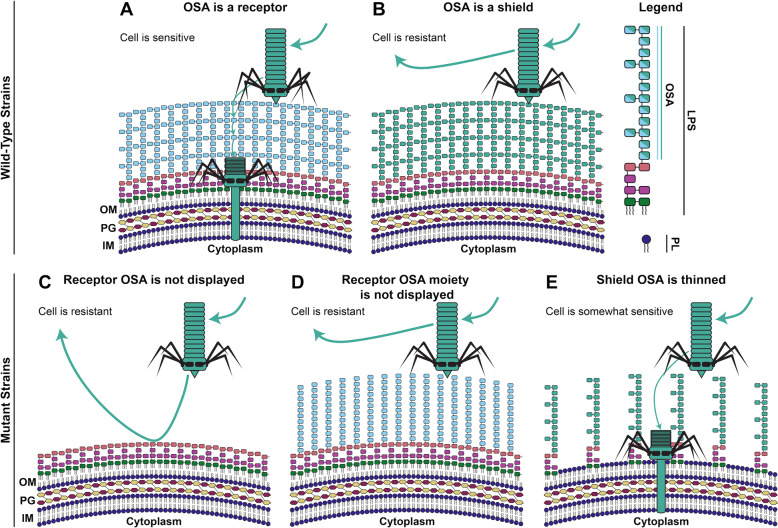
Proposed effect of O-specific antigens on tailocin lethality. We summarize our high-throughput reverse genetics data to propose five different OSA display scenarios that define whether a strain is sensitive or resistant to a tailocin. **A** Strain displays an LPS molecule whose OSA can be bound by the tailocin (is a receptor). The strain is naturally sensitive. **B** Strain displays an LPS molecule whose OSA interferes with tailocin binding (is a non-receptor/shield). The strain is naturally resistant. **C** Mutation in OSA biosynthesis/display leads to inability of the mutant to display receptor OSA. The mutant has become resistant. **D** Mutation in OSA biosynthesis/display leads to inability of the mutant to display the specific receptor OSA moiety. The mutant has become resistant. **E** Mutation in retrograde phospholipid transport or anterograde LPS transport leads to a thinning of the mutant’s shield LPS. The mutant has become more sensitive to tailocins. OM outer membrane, PG peptidoglycan, IM inner membrane, OSA O-specific antigen, LPS, lipopolysaccharide, PL phospholipid.

We summarize the major genetics findings from this study by proposing five scenarios that illustrate the effect of OSA on tailocin sensitivity (Fig. 5). Our data support a model put forward by Köhler et al. [12] that LPS—or, specifically, the OSA—acts as either a receptor for, or shield against, specific tailocins [35, 37, 38]. Whether a specific OSA molecule serves as a receptor or shield for a specific tailocin particle is determined by the chemical structure of the OSA molecule and the morphology of the tailocin receptor-binding protein(s). When an OSA molecule serves as a receptor, it can be bound tightly by the tailocin, recruiting it toward the cell envelope, increasing the likelihood of lethal penetration, and resulting in increased sensitivity. On the other hand, a shield OSA molecule cannot be bound by the tailocin, and acts as a physical barrier between the tailocin and the cell envelope, resulting in increased resistance. Mutations resulting in removal of a receptor OSA, or just the receptor moiety of the OSA, can make a sensitive strain resistant to a specific tailocin. Alternatively, if the strain possesses a shield OSA against the tailocin, that shield can be weakened by thinning the density of OSA at the cell envelope, in turn increasing sensitivity to tailocins. In other words, the composition and density of the LPS can help protect bacteria from tailocins, just as it protects them from small molecule antimicrobials and phage infections [53].

Assumptions made under this model (Fig. 5) include: (1) uncapped LPS alone can act as a sufficient shield against our tailocins; and (2) tailocins can kill any strain, so long as they can get close enough to the cell envelope to penetrate completely. The first assumption is based on evidence that many phages (including HK620, P22, Sf6, 9NA, and Det7) are unable to infect strains displaying an uncapped (OSA null) LPS [54]. The second assumption is made from the observation that wild-type Pse06, which is “resistant” to its own tailocins, still displays an impaired OD_600_ growth curve in the presence of those tailocins (Fig. 3C). This observation could be explained by low-frequency killing of the producing strain by its own tailocins should they spontaneously pass through the OSA shield and contract close enough to the cell envelope. Thus, we perhaps need to be careful how we use the terms “sensitive” and “resistant”, which are assigned upon the presence or absence respectively of a zone of inhibition during a spot assay. Instead, intermediary phenotypes may be common, and may depend on genetic and temporal variation in LPS display and packing [35]. Acquisition of more nuanced data could be achieved through widespread use of growth curves in assessing tailocin sensitivity in future.

Since our RB-TnSeq fitness assays yield a small set of very high scoring genes, identification of the most important factors involved in tailocin sensitivity is straightforward. However, strong positive selection for mutations in these genes shrouds our ability to detect factors with weaker impact on tailocin sensitivity. Mutants with lower relative fitness are rapidly outcompeted from the population and become undetectable in our data. Furthermore, we recognize that RB-TnSeq limits us to the study of non-essential genes. Many genes that modify the cell envelope (e.g., LPS and PL biosynthetic and transport genes) are essential, as noted in Figs. 2C and 3B. We were unable to evaluate the impact of these and other essential genes on tailocin sensitivity in this study. Future studies could improve on this using complementary mutational approaches [55, 56] that have recently been applied to investigate the genetic determinants of phage sensitivity [32].

Overall, our fitness data suggest that tailocin sensitivity factors are limited to those affecting LPS composition and display, and can be found at well-defined genomic loci known to influence these phenotypes. The fitness data specifically emphasize the importance of the OSA. However, this assertion is somewhat disputed by our high-throughput tailocin sensitivity phenotyping data, in which a subset of strains can vary in tailocin sensitivity despite sharing very similar OSA biosynthetic clusters. In our SM strains, differences in tailocin sensitivity cannot be explained by nucleotide variation in the OSA cluster (Supplementary Fig. S9), *mla* or *lpt* genes, or by GWAS analysis. Thus, the responsible factors may be hard to discern at the sequence level, or may have resulted from sequence changes after we sequenced the strains. Gammaproteobacteria are known to escape phage predation through reversible and heritable (i.e., phase variable) changes in the expression of LPS-modifying proteins [57–62]. It is possible that similar mechanisms are at work in our SM strains. In summary, the genotypes of tailocin sensitivity are more complicated than just the presence/absence of LPS-modifying genes.

This study represents the most comprehensive examination of tailocin sensitivity determinants to date. We assigned new experimental tailocin sensitivity phenotypes to orthologs of both well-studied and hypothetical genes, highlighting the efficacy of our methods for studying non-model bacteria. Expanding on earlier work [38], we also performed the first genome-wide investigation of resistance to tailocin-mediated self-intoxication and indicate the importance of LPS density in protecting a strain from its own tailocins. Our findings add weight to a model that LPS acts as either a receptor for, or a shield against, tailocins [12], and provide a framework for studying bacterial evolution in the context of tailocin-mediated interference competition. Since LPS plays a crucial role in tailocin-mediated killing and self-intoxication, investigation of tailocin sensitivity across biotic and abiotic conditions that affect LPS architecture could provide valuable information on interference competition in diverse contexts.

## Methods

### Bacterial strains, oligos, plasmids, and growth conditions

Strains used in this study are described in Supplementary Table S1, oligos in Supplementary Table S23, plasmids in Supplementary Table S24, and RB-TnSeq mutant libraries in Supplementary Table S5. The method for mutant library construction is described by Wetmore et al. [21], and the specific libraries we employed in this study are described by Price et al. [22]. All cultures were aerobic. LB-Lennox (LB-L) media [63] (Sigma-Aldrich) was used to cultivate all bacteria. *E. coli* and *P. aeruginosa* were incubated at 37 °C. *P. fluorescens*, *P. syringae*, and groundwater *Pseudomonas* isolates were incubated at 30 °C. Liquid cultures were shaken at 200 rpm, except for 96-well plate cultures that were shaken at 750 rpm. When appropriate, antibiotics were supplemented to LB-L cultures at the following concentrations: carbenicillin at 100 µg/mL (LB-L-Cb), kanamycin at 50 µg/mL (LB-L-Km).

### Strain isolation

All environmental strains described in this study were isolated from groundwater wells at the Oak Ridge Field Research Center in Oak Ridge, TN. We used 1 mL aliquots of groundwater to inoculate various types of liquid media for growth. Positive growth was identified by an increase in culture turbidity. Positive cultures were sequentially transferred in the same media twice, before streaking onto agar plates for single colonies. Individual colonies were picked and restreaked again to check purity. For storage, axenic colonies were regrown in liquid media to mid-log phase, amended with sterile glycerol to a final concentration of 30%, flash frozen with liquid nitrogen, and stored at −80 °C. The groundwater well, media, and growth conditions used to isolate each strain can be found in Supplementary Table S1 [22, 64–67]. While a variety of aerobic and anaerobic enrichment strategies were used for isolation, all strains are capable of growth in LB-L aerobically.

### Genome sequencing

For genomic DNA extraction, isolates were revived from glycerol stocks into 500 µL liquid LB-L media in wells of 2.0 mL 96-well DeepWell™ blocks (ThermoFisher™ Nunc™). The blocks were incubated with shaking until the cultures reached stationary phase. Cells were pelleted by centrifugation (3220 g, 5 min) and the supernatant was decanted. Genomic DNA was manually purified using the QIAamp 96 DNA QIAcube HT kit (QIAGEN) and a vacuum manifold. All samples were eluted in AE buffer (10 mM Tris-HCl, 0.5 mM EDTA, pH 9.0). The samples were then randomly plated into a 384-well plate for automated Illumina library preparation. Each genomic DNA sample was normalized to 0.2 ng/µL in 10 mM Tris (pH 8.0). Then, libraries were prepared using the Nextera XT kit (Illumina) at 1/12th reaction size with a TTP LabTech mosquito^®^ HV liquid handling robot. Final libraries were cleaned with SPRIselect beads (Beckman Coulter) and pooled before sequencing on an Illumina NextSeq 500 producing 2 × 150 bp paired-end reads. Separately, genomic DNA from the 12 Pse## codenamed strains (Supplementary Table S1) was library prepped and sequenced on a PacBio Sequel with P5C3 chemistry at the DNA Technologies & Expression Analysis Core Laboratory at UC Davis. Standard protocols were used for PacBio library prep and the PacBio reads were combined with Illumina reads for assembly.

### Genome assembly

Reads were preprocessed using BBtools version 38.60 to remove Illumina adapters, perform quality filtering and trimming, and remove PhiX174 spike-ins. We are not aware of any published papers documenting these tools. However, it is a standard tool suite developed at the Department of Energy Joint Genome Institute and is documented at https://jgi.doe.gov/data-and-tools/bbtools/. Processing was done in two passes. First bbduk.sh was run with parameters *ktrim* = *r k* = *23 mink* = *11 hdist* = *1 ref* = *adapters.fa tbo tpe 2*. This was to remove any remaining Illumina adapters given in adapters.fa (standard Illumina adapters). Then bbduk.sh was run again with parameters *bf1 k* = *27 hdist* = *1 qtrim* = *rl trimq* = *17 cardinality* = *t ref* = *phix174_Illumina.fa*. This was to perform quality filtering and trimming as well as remove Illumina PhiX174 spike-ins given in the file phix174_Illumina.fa. Assembly was performed using SPAdes version 3.13.0 [68] with parameters *-k 21,33,55,77,99,127*.

### Identification of tailocin clusters

Tailocin elements were identified in assemblies of the studied isolates and in selected complete *Pseudomonas* spp. genomes using the PHASTER webserver [69]. Groups of orthologous genes (orthogroups) were identified using OrthoFinder 2.0 [70]. Insertion sites for tailocin loci were identified by searching for orthologs of *mutS, cinA*, *trpE*, and *trpG* genes. A locus between *mutS* and *cinA* was considered tailocin-encoding if PHASTER identified it as an incomplete prophage. No tailocin-encoding genes were found between *trpE* and *trpG* genes in any of the studied isolates. Functional annotations for tailocin genes were determined by transfer from orthologous tailocin genes previously characterized in *P. chlororaphis* [30], *P. putida*, and *P. aeruginosa* [25]. For genes lacking characterized orthologs, functions were assigned in accordance with PHASTER annotations. Tailocin clusters were visualized using the R package “genoPlotR” [71].

### Induction and purification of tailocins

5 mL *P. fluorescens* LB-L cultures were cultivated overnight. These were then back-diluted 1:100 in 100 mL LB-L and incubated with shaking in baffled 500 mL Erlenmeyer flasks until OD_600_ reached ~0.5. At this point, mitomycin C was applied to a final concentration of 0.5 µg/mL to induce tailocin production, then incubation with shaking was resumed for an additional ~18 h. 10 µL chloroform was added to lyse remaining intact cells. Cultures were centrifuged (3220 g, 1 h, 4 °C) to pellet cell debris. The supernatants were then sterilized by filtration (0.22 µm filter). Ammonium sulfate was added to 30% saturation (16.4 g/100 mL), and dissolved by stirring with a magnetic stir bar in the cold room for 30 min. Precipitate was collected by centrifugation (16,000 g, 37.5 min, 4 °C). The supernatant was decanted, and the precipitate was resuspended in 4 mL SM buffer (100 mM NaCl, 8 mM MgSO_4_•7H_2_O, 50 mM Tris-Cl, 0.01% gelatin) and stored at 4 °C. Uninduced control samples were prepared in the same way as experimental samples except that mitomycin C addition was omitted.

### Low-throughput spot test phenotypic assay

25 × 150 mm glass culture tubes of 3 mL liquid LB-L media were each inoculated with a different target strain from a frozen glycerol stock. The tubes were incubated with shaking until the cultures reached stationary phase. In the meantime, 100 × 15 mm Petri dishes (BD Biosciences) were prepared with ~25 mL standard LB-L (1.5%) agar and cooled. 200 µL of each stationary phase culture was mixed with 5 mL molten LB-L soft (0.5%) agar, then immediately decanted over a pre-poured 1.5% LB-L agar plate with care taken to cover the entire surface. The plates were allowed to cool for 30 min at room temperature. 2 µL stock or serially diluted tailocin sample was pipetted onto the solidified soft agar, then allowed to dry for 10 min. The plates were then incubated, and the interaction was deemed sensitive if a zone of inhibition was observed within 40 h. When testing serially diluted tailocin samples, no plaques were observed, only zones of inhibition. This indicates that the killing agent is nonreplicative, and thus not reminiscent of a phage. When testing uninduced tailocin control samples (prepared without mitomycin C application) no lethality was observed (data not shown). Spot tests were performed in biological triplicates.

### Higher-throughput spot test phenotypic assay

Wells of 2.0 mL 96-well DeepWell™ blocks (ThermoFisher™ Nunc™) were filled with 1 mL liquid LB-L media, then each well was inoculated with a different target strain from a frozen glycerol stock. The blocks were incubated with shaking until the cultures reached stationary phase. In the meantime, 48-well Bio-One CELLSTAR^®^ plates (Greiner) were prepared with 800 µL standard LB-L (1.5%) agar in each well, and cooled. 6 µL of each stationary phase culture was mixed with 160 µL molten LB-L soft (0.5%) agar, then immediately pipetted into a well of a pre-prepared 1.5% LB-L agar 48-well plate with care taken to cover the entire surface. The plates were allowed to cool for 30 min at room temperature. 2 µL stock or serially diluted tailocin sample was pipetted onto the soft agar, then allowed to dry for 10 min. The plates were then incubated, and the interaction was deemed sensitive if a zone of inhibition was observed within 40 h.

### Transmission electron microscopy (TEM)

TEM was performed at Donner Laboratory at Lawrence Berkeley National Laboratory. 4 µL of tailocin samples, purified as above, was placed on glow-discharged carbon-coated grids (Formvar-carbon, 200 mesh copper, Electron Microscopy Sciences) for 5 min, then partially blotted to ~1 µL. Grids were then placed sample side down on a drop of ddH_2_O for 5 min, then transferred to a second drop of ddH_2_O for another 5 min. Residual water was partially blotted and 3 µL of 2% aqueous uranyl acetate was applied. After 2 min, the sample was blotted to dryness. Grids were visualized on a JEOL JEM-1200× electron microscope operating at an accelerating voltage of 80 kV. Images were recorded at 30,000× and 60,000× magnification with a charge-coupled-device camera (UltraScan^®^, Gatan). Image analysis was done using ImageJ.

### Protein mass spectrometry

Tailocins purified as above were subjected to tryptic digestion and carboxyamidomethylation of cysteines performed in 40% (v/v) methanol, 5 mM TCEP, 100 mM ammonium bicarbonate, at pH 8.5. Mass spectrometry was then performed by the UC Berkeley Vincent J. Coates Proteomics/Mass Spectrometry Laboratory. MudPIT methods were used in order to achieve good sequence coverage of target proteins in a complex mixture [72]. A nano-LC column was packed in a 100 µm inner diameter glass capillary with an emitter tip. The column consisted of 10 cm of Polaris c18 5 μm packing material (Varian), followed by 4 cm of Partisphere 5 SCX (Whatman). The column was loaded by use of a pressure bomb and washed extensively with buffer A (5% acetonitrile and 0.02% heptafluorobutyric acid). The column was then directly coupled to an electrospray ionization source mounted on a ThermoFisher LTQ XL linear ion trap mass spectrometer. An Agilent 1200 HPLC equipped with a split line so as to deliver a flow rate of 300 nL/min was used for chromatography. Peptides were eluted using a four-step MudPIT procedure [72].

### Analysis of mass spectrometry data

Protein identification was done using the Integrated Proteomics Pipeline (IP2, Integrated Proteomics Applications, Inc. San Diego, CA) using ProLuCID/Sequest, DTASelect2 and Census [73–76]. Tandem mass spectra were extracted into ms1 and ms2 files from raw files using RawExtractor [77]. Data were searched against a database of protein sequences specific for each sample supplemented with sequences of common contaminants and concatenated to a decoy database in which the sequence for each entry in the original database was reversed [78]. LTQ data were searched with 3000.0 mmu precursor tolerance and the fragment ions were restricted to a 600.0 ppm tolerance. All searches were parallelized and searched on the Vincent J. Coates proteomics cluster. Search space included all fully tryptic peptide candidates with no missed cleavage restrictions. Carbamidomethylation (+57.02146) of cysteine was considered a static modification. We required one peptide per protein and both tryptic termini for each protein identification. The ProLuCID search results were assembled and filtered using the DTASelect program [74, 75] with a peptide false discovery rate (FDR) of 0.001 for single peptides and a peptide FDR of 0.005 for additional peptides for the same protein. The molar percentage of protein content for each predicted tailocin protein was calculated from exponentially modified protein abundance index (emPAI) [79] as $\frac{{\rm{emPAI}}}{{\Sigma (\rm{emPAI})}} \times 100$ and is listed in Supplementary Table S4. Overall, the following proportions of each tailocin sample are tailocin proteins: Pse04 Tailocins (15.6%); Pse05 Tailocins (9.4%); Pse06 Tailocins (7.7%), Pse11 Tailocins (14.6%).

### Competitive mutant fitness assays

We performed all our assays in 48-well microplates using the assay design, culture, and data collection settings described previously [21]. In our assays, our partially purified tailocins were used as stressors at final concentrations 0.5× or 0.05× of the stock preparation. SM buffer was supplied as a tailocin-free control. We supplied LB-L (for wild-type strains) or LB-L-Km (for mutants) as the growth medium in each case. The microplates were incubated in a Tecan Infinite F200 plate reader with orbital shaking and OD_600_ readings every 15 min. Mutant library cultures were harvested for subsequent BarSeq analysis at mid-log phase, as determined from OD_600_ traces. Cell pellets were obtained by centrifugation (8000 g, 3 min) and stored at −20 °C awaiting genomic DNA extraction. Each condition was assayed in duplicate.

### BarSeq

Genomic DNA extraction and barcode PCR were performed as described previously [21, 22]. All genomic DNA extractions were done using the DNeasy Blood and Tissue kit (QIAGEN) and quantified using a NanoDrop 1000 device (Thermo Fisher). Thermocyclers were set to the 98 °C BarSeq PCR protocol (“BarSeq98”) [21]. A primer set that permits improved cluster discrimination on the Illumina HiSeq 4000 was used (Supplementary Table S23) [22]. Barcode sequence data were obtained by multiplexing samples on a lane of a HiSeq 4000 (50-cycle single read) run at the UC Berkeley Vincent J. Coates Genomics Sequencing Laboratory. Fitness data were calculated and analyzed from BarSeq reads as previously described [21].

### Analysis of BarSeq data

BarSeq data were analyzed as described previously [21]. Then, fitness hits were filtered further as in our phage resistance studies [32, 33] to reduce false positives caused by a lack of coverage of most mutants following stringent tailocin selection. To be evaluated as a positive fitness hit, gene disruptions had to exhibit a fitness score ≥7.0 (Supplementary Tables S7, S10, and S13), a *t*-like statistic ≥5.0 (Supplementary Tables S8, S11, and S14), and show an increase in barcode read count from before treatment (Time0) to after treatment (Supplementary Tables S6, S9, and S12). To be evaluated as a negative fitness hit, gene disruptions had to exhibit a fitness score ≤−2.0 (Supplementary Table S19), a *t*-like statistic ≤−5.0 (Supplementary Table S20), and show a decrease in barcode read count from before treatment (Time0) to after treatment (Supplementary Table S18). Heatmaps of fitness scores were generated using Morpheus (https://software.broadinstitute.org/morpheus).

### Validation of tailocin resistance phenotypes

Individual transposon-insertion mutants for validating tailocin resistance phenotypes were isolated by enriching them from their respective pooled RB-TnSeq libraries through the application of tailocins that the wild-type strain is sensitive to. RB-TnSeq libraries, at a starting OD_600_ of 0.02, were cultured with tailocins at 0.5× stock concentration, in the wells of a 96-well microplate (200 µL per well). The microplates were incubated in a Tecan Infinite F200 plate reader with orbital shaking and OD_600_ readings every 15 min. Cultures were halted at early-log phase and spread on LB-L-Km agar for single colonies. Single colonies were subcultured in LB-L-Km, then subjected to colony arbitrary PCR to determine barcode and transposon-insertion site. A description of the colony arbitrary PCR is as follows. 100 µL of saturated culture was pelleted, resuspended in 2.5% v/v IGEPAL^®^ CA-630 (Sigma-Aldrich) and boiled at 98 °C for 20 min. Cell debris was pelleted by centrifugation. 1 µL of supernatant was then used as template for the first round of arbitrary PCR: a 20 µL total volume Q5^®^ Hot Start polymerase reaction (New England Biolabs) with 2.5 pmol oALA051, 2.5 pmol oALA052, and 5 pmol oALA054 primers (see Supplementary Table S23 for primer sequences). First round arbitrary PCR thermal cycling conditions were: 98 °C for 3 min; 5 cycles of 98 °C for 30 s, 42 °C (−1 °C/cycle) for 30 s, 72 °C for 3 min; 25 cycles of 98 °C for 30 s, 55 °C for 30 s, 72 °C for 3 min; 72 °C for 5 min. 2 µL of first round PCR product was used as template for the second round of arbitrary PCR: 20 µL total volume Q5^®^ Hot Start polymerase reaction with 2.5 pmol oALA053, and 2.5 pmol oALA055. Second round arbitrary PCR thermal cycling conditions were: 98 °C for 3 min; 30 cycles of 98 °C for 30 s, 55 °C for 30 s, 72 °C for 3 min; 72 °C for 5 min. 10 µL second round arbitrary PCR product was cleaned using AMPure beads (Beckman Coulter) and Sanger sequenced using oALA055. 14 tailocin-resistant single strains were identified and isolated in this way (see Supplementary Tables S1 and S15). Tailocin resistance was verified by spot test phenotypic assay (see above).

### Validation of tailocin production phenotypes

Tailocin production and tailocin sensitivity phenotypes were validated through marked deletions of baseplate genes and genes with significant negative fitness scores, respectively. To do this, we assembled derivatives of the plasmid pMO7704 to generate replacements of our genes of interest with a kanamycin resistance marker via double crossover. Plasmid assembly and strain construction details can be found in Supplementary Tables S24 and S1, respectively. Plasmids propagated in the *E. coli* 10-beta cloning strain were purified using the standard QIAprep protocol (QIAGEN), then delivered to the *Pseudomonas* strain via previously published electroporation [80] or conjugation [81] protocols. Transformants were selected on LB-L-Km agar. Disruption of tailocin production was verified by spot test phenotypic assay (see above).

### Validation of tailocin sensitivity phenotypes

Increases in tailocin sensitivity were too subtle to be detected by spot assay (data not shown). Instead, sensitivity was verified by comparing growth in planktonic culture of mutants vs. wildtype in the presence of tailocins. Cultures were prepared at a starting OD_600_ of 0.02, with tailocins added at 0.5× stock concentration, in the wells of a 96-well microplate (200 µL total volume per well). The microplates were incubated in a Tecan Infinite F200 plate reader with orbital shaking and OD_600_ readings every 15 min.

### Identification and comparison of lipopolysaccharide biosynthetic gene clusters

Gene clusters encoding LPS core oligosaccharide, OSA, and CPA biosynthetic enzymes were identified by analysis of orthologous groups of genes (orthogroups) as described above, using OrthoFinder 2.0 [70]. Orthologs of *P. aeruginosa* PAO1 genes PA4997-PA5012 and PA5447-PA5459 were considered to represent LPS core oligosaccharide and CPA gene clusters, respectively, in accordance with existing data on gene functions [34]. As gene content of OSA biosynthetic loci is variable, highly conserved flanking genes were used to determine locus boundaries: *himD*/*ihfB* on the 5’ end and *wbpM* on the 3’ end [82]. All genes located between these two genes were considered to form OSA biosynthetic clusters. Gene clusters were visualized using the R package “genoPlotR” [71]. To compare gene contents of OSA clusters, Jaccard distances for all pairs of strains were computed as 1 minus the number of orthogroups present in OSA clusters of both strains divided by the number of orthogroups present in either of the OSA clusters. The resulting matrix was used to compute a hierarchical cluster using the “hclust” function in R (v3.0.2) (method = “single”) and visualized with the “dendextend” package in R and the Interactive Tree of Life (iTOL) online tool [83].

### Phylogenetic analysis

To estimate phylogenetic relationships between genomes of our collection of *Pseudomonas* spp. strains, we identified a set of 120 bacterial marker genes with GTDB-Tk toolkit [23]. Only 88 marker genes were found in single copy in each of the 130 genomes studied. Gene sequences of those 88 markers were aligned by MAFFT v7.310 [84] with *--auto* option, and the resulting 88 alignments were concatenated into a single multiple sequence alignment. A phylogenetic tree was reconstructed from the multiple alignment using the maximum likelihood method implemented in the FastTree software v2.1.10 [85] using the generalized time-reversible (-gtr) model and visualized using the iTOL online tool [83].

## Supplementary information

Supplementary Material

Supplementary Tables
