# Supplementary material for: Systematic discovery of pseudomonad genetic factors involved in sensitivity to tailocins
Source: ISME J. 2021 Mar 1;15(8):2289–305. doi: 10.1038/s41396-021-00921-1 (PMC8319346; doi:10.1038/s41396-021-00921-1)
Supplement: Supplementary file 1 — Supplementary Material [file 41396_2021_921_MOESM1_ESM.docx]

# Supplementary Information for

Systematic Discovery of Pseudomonad Genetic Factors Involved in Sensitivity to Tailocins

Sean Carim^1^, Ashley L. Azadeh^2^, Alexey E. Kazakov^3^, Morgan N. Price^3^, Peter J. Walian^4^, Lauren M. Lui^3^, Torben N. Nielsen^3^, Romy Chakraborty^5^, Adam M. Deutschbauer^1,3^, Vivek K. Mutalik^3,6*^, Adam P. Arkin^3,6,7*^

^1^Department of Plant and Microbial Biology, University of California, Berkeley, CA

^2^Innovative Genomics Institute, University of California, Berkeley, CA

^3^Environmental Genomics and Systems Biology, Lawrence Berkeley National Laboratory, Berkeley, CA

^4^Molecular Biophysics and Integrated Bioimaging, Lawrence Berkeley National Laboratory, Berkeley, CA

^5^Climate and Ecosystems Sciences, Lawrence Berkeley National Laboratory, Berkeley, CA

^6^Innovative Genomics Institute, Berkeley, CA

^7^Department of Bioengineering, University of California, Berkeley, CA

^*^To whom correspondence should be addressed:

[VKMutalik@lbl.gov](mailto:VKMutalik@lbl.gov); [APArkin@lbl.gov](mailto:APArkin@lbl.gov)

# Supplementary Information

**Table of Contents**

# Supplementary Figures

- Fig. S1. Extended tailocin gene clusters.
- Fig. S2. Initial tailocin susceptibility data.
- Fig. S3. Tailocin production phenotype validations.
- Fig. S4. Tailocin resistance phenotype validations.
- Fig. S5. LPS core oligosaccharide biosynthetic gene clusters.
- Fig. S6. O-specific antigen biosynthetic gene clusters.
- Fig. S7. Tailocin sensitivity phenotype validations.
- Fig. S8. Phylogenetic tree of target strains overlaid with tailocin sensitivity data.
- Fig. S9. O-specific antigen biosynthetic gene clusters of target strains overlaid with tailocin sensitivity data.

**Supplementary Notes**

## Supplementary Note 1. Review of the roles of LPS inner core biosynthetic enzymes on O-specific antigen display

## Supplementary Note 2. Putative functions of AO361_RS10865 and AO361_RS10900, genes involved in sensitivity to a subset of antagonistic tailocins

**Supplementary Tables** (in separate .xlsx workbook)

1. Table S1. Bacterial strains used in this study.
2. Table S2. Tailocin genes identified via our bioinformatics approach.
3. Table S3. Tailocin gene orthogroups across *Pseudomonas* strains.
4. Table S4. Proteomics results for tailocin samples.
5. Table S5. Description of RB-TnSeq mutant libraries used in this study.
6. Table S6. Read count data for Pse05 genes implicated in tailocin sensitivity.
7. Table S7. Additional fitness data for Pse05 genes implicated in tailocin sensitivity.
8. Table S8. t-like statistics data for Pse05 genes implicated in tailocin sensitivity.
9. Table S9. Read count data for Pse03 genes implicated in tailocin sensitivity.
10. Table S10. Additional fitness data for Pse03 genes implicated in tailocin sensitivity.
11. Table S11. t-like statistics data for Pse03 genes implicated in tailocin sensitivity.
12. Table S12. Read count data for Pse13 genes implicated in tailocin sensitivity.
13. Table S13. Additional fitness data for Pse13 genes implicated in tailocin sensitivity.
14. Table S14. t-like statistics data for Pse13 genes implicated in tailocin sensitivity
15. Table S15. Descriptions of all enriched transposon-insertion mutants used for validations.
16. Table S16. LPS gene orthogroups across *Pseudomonas* strains.
17. Table S17. O-specific antigen gene orthogroups across 13 *Pseudomonas* strains.
18. Table S18. Read count data for Pse06 genes implicated in tailocin resistance.
19. Table S19. Additional fitness data for Pse06 genes implicated in tailocin resistance.
20. Table S20. t-like statistics data for Pse06 genes implicated in tailocin resistance.
21. Table S21. Killing activity of tailocin samples across 130 *Pseudomonas* strains.
22. Table S22. O-specific antigen gene orthogroups across 130 *Pseudomonas* strains.
23. Table S23. Oligonucleotide used in this study.
24. Table S24. Plasmids used in this study.

**
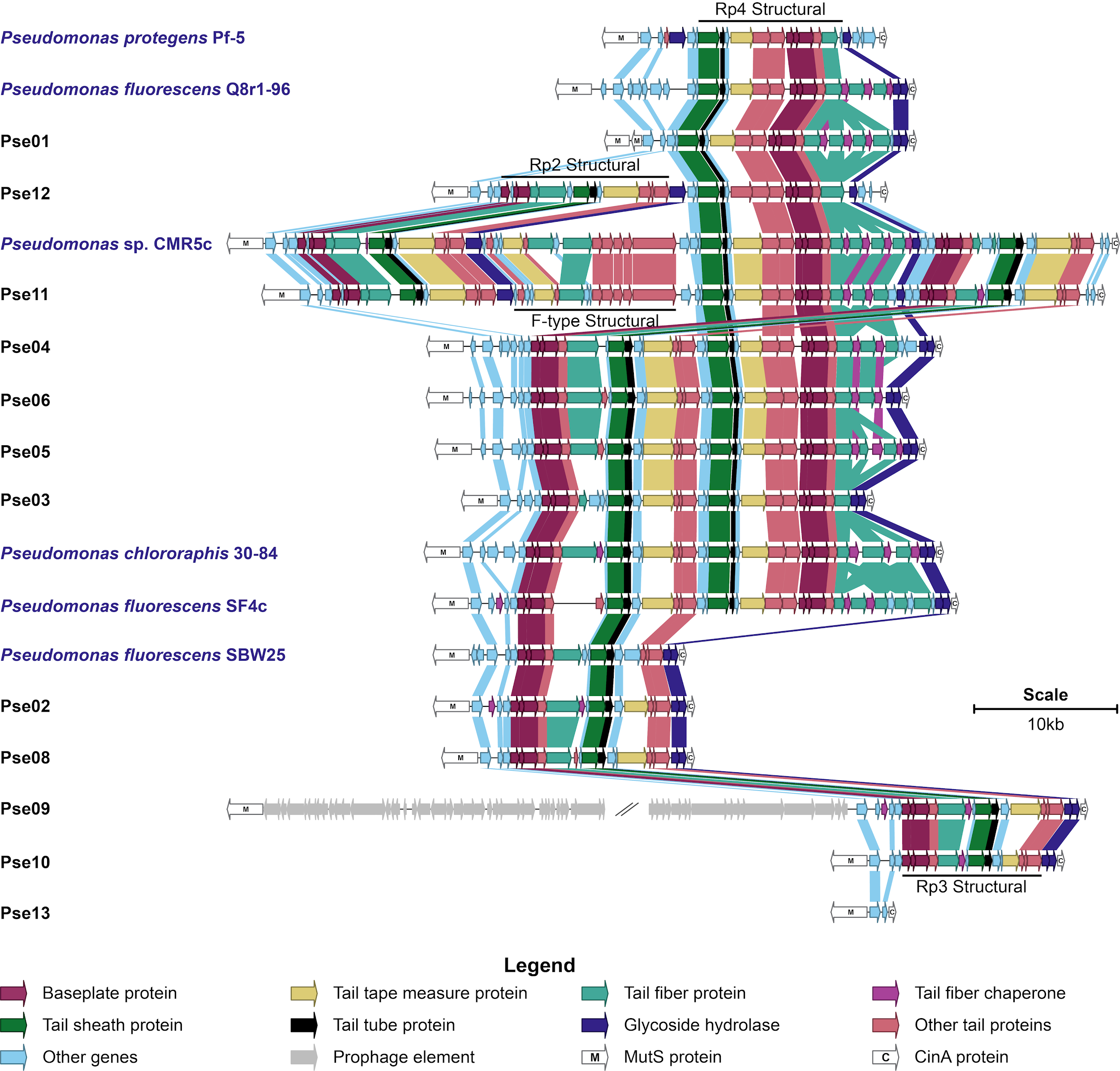
**

### Figure S1. Extended tailocin gene clusters. Tailocin gene clusters of various sizes were found in 11 of our 12 selected *Pseudomonas* isolates, and solely at the *mutS*/*cinA* locus. Pse09 appears to encode a full prophage of unknown length at this locus in addition to an Rp3 tailocin cluster. Pse13 has no tailocin gene cluster. Tailocin gene clusters in previously studied soil and rhizosphere *Pseudomonas* strains (Pf-5, Q8r1-96, CMR5c, 30-84, SF4c, SBW25), labeled in blue text, are included for comparison. Genes are colored by the presence of key words in their PHASTER[^30^](https://paperpile.com/c/gPnyZb/UDeWg) predicted annotations. Genes in the same orthogroup (Methods) are joined by a block of color. Additional data on these clusters can be found in Table S2. For a full list of orthogroups, see Table S3.

**
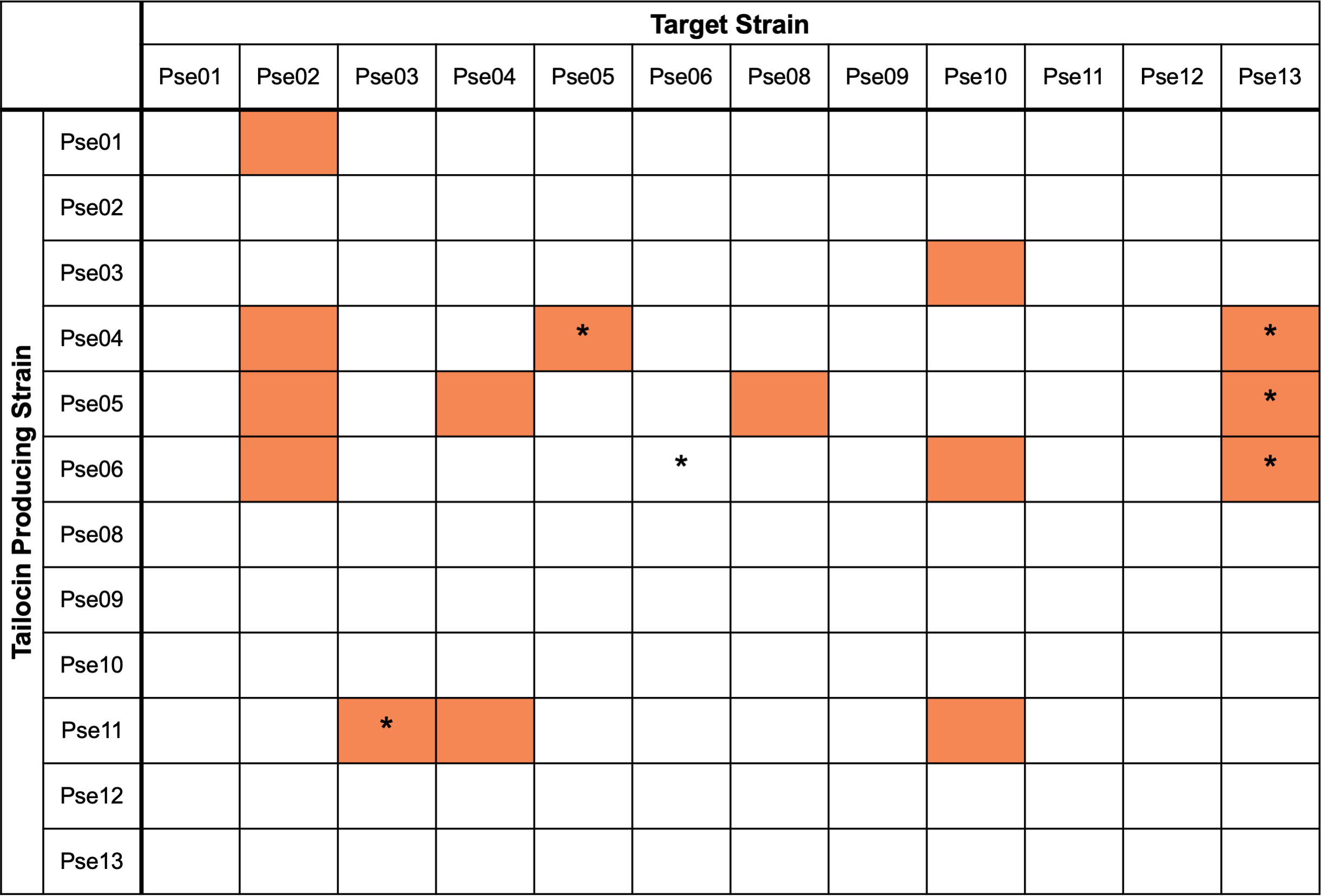
**

### Figure S2. Initial tailocin susceptibility data. Partially purified tailocin samples from 12 *Pseudomonas* strains were used to challenge the same 12 strains by spot testing. Note: Pse13 does not encode tailocins, so we did not expect its ‘tailocin sample’ to be lethal to any target strain. Orange and white highlighted cells illustrate sensitive and resistant interactions respectively. No tailocin sample killed its producing strain. Starred (*) cells illustrate interactions that were investigated in greater depth due to the availability of RB-TnSeq[^22^](https://paperpile.com/c/gPnyZb/g2xkC) mutant libraries in the target strains.

**
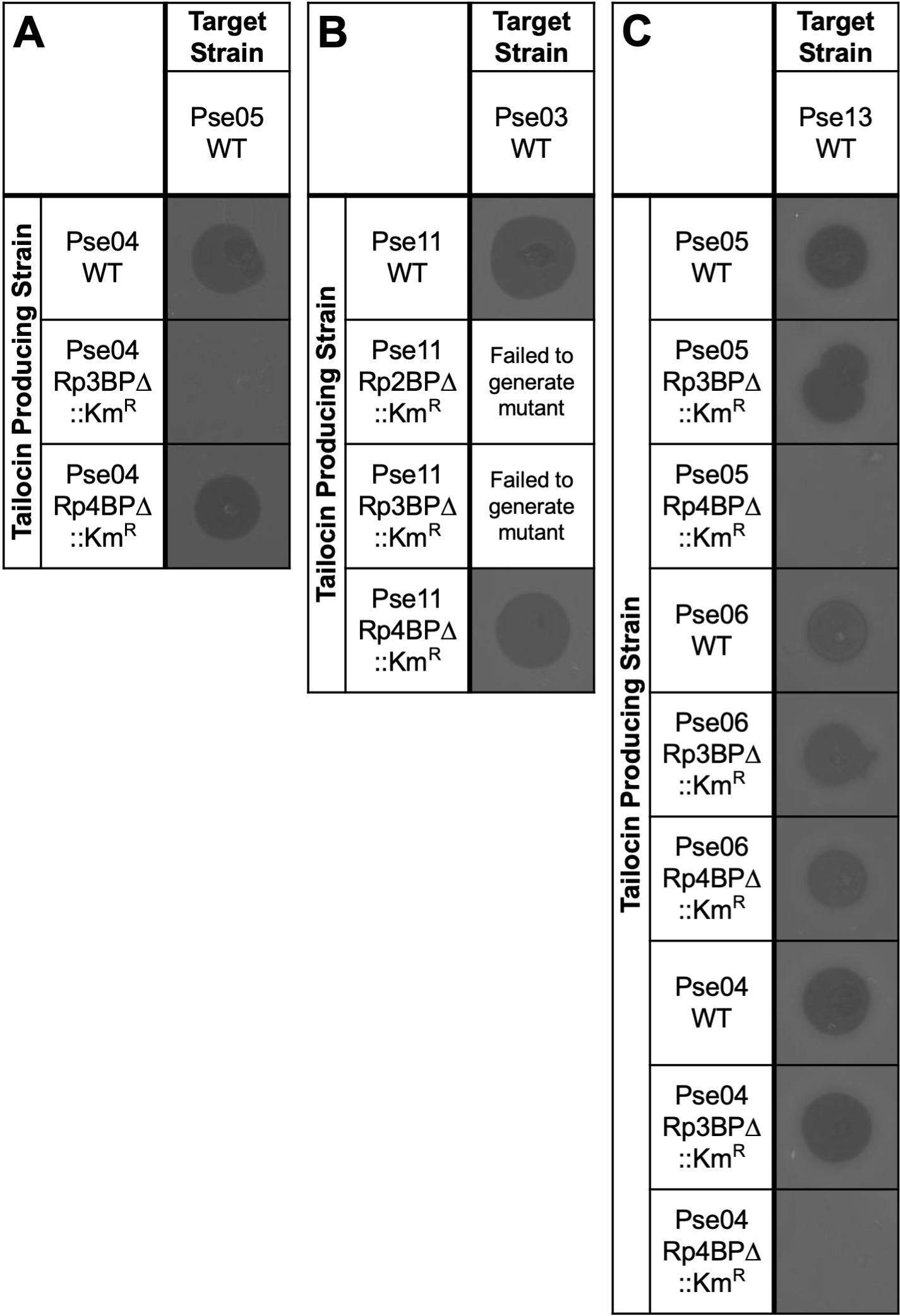
**

### Figure S3. Tailocin production phenotype validations. Marked gene deletion vectors were used to replace baseplate protein genes with a kanamycin-resistance marker. Tailocins were induced and partially purified from these baseplate mutants and spotted on strains sensitive to the wild-type tailocin. Target strains are: (A) Pse05; (B) Pse03; (C) Pse13. BP, baseplate genes. This data suggests that the Pse04 Rp3 tailocin is responsible for killing Pse05, while its Rp4 tailocin in turn kills Pse13. Meanwhile, only the Rp4 tailocin of Pse05 appears to kill Pse13. Finally, both tailocin particles of Pse06 kill Pse13. We were unable to generate Rp2BP or Rp3BP mutants in Pse11.

**
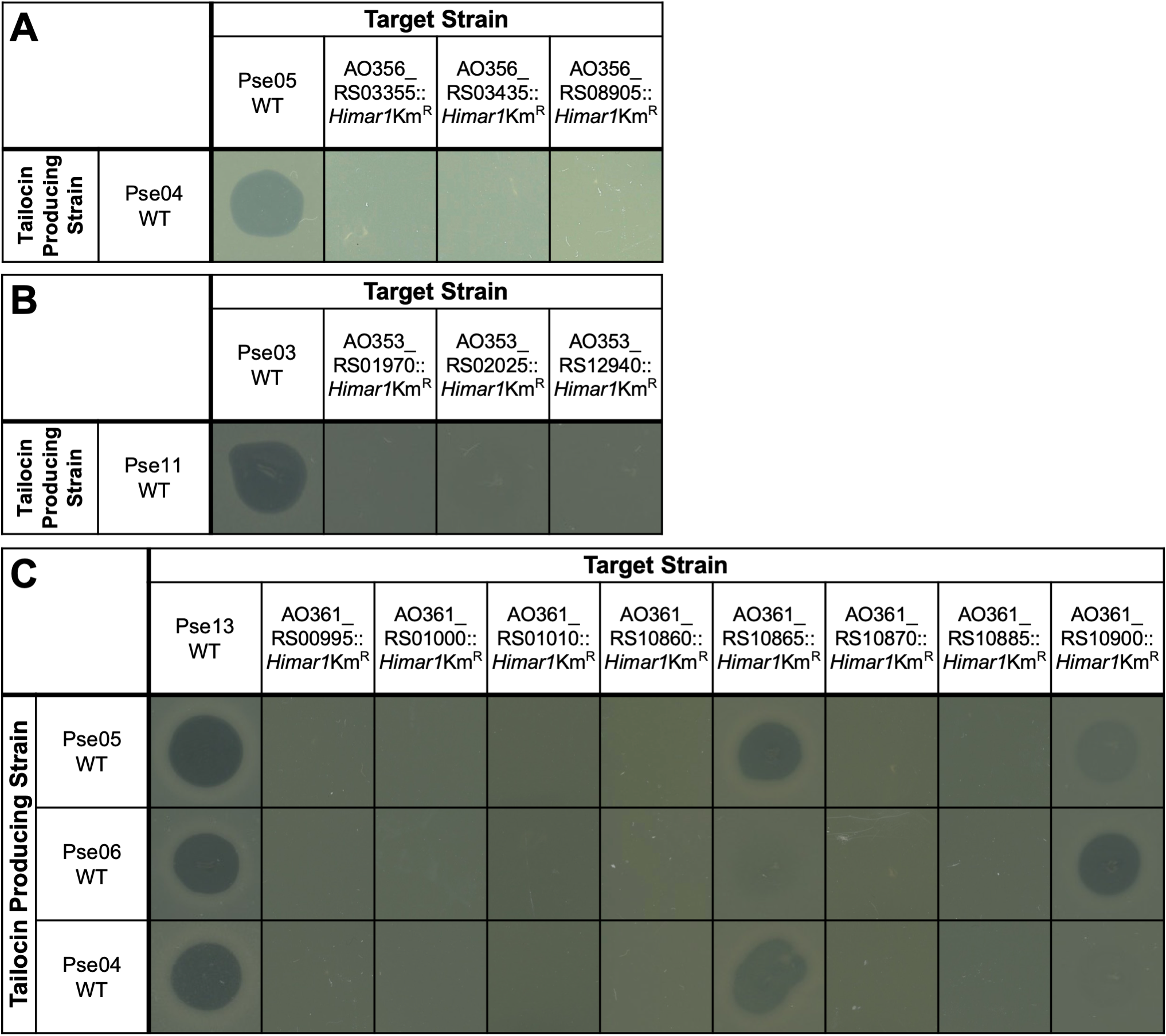
**

### Figure S4. Tailocin resistance phenotype validations. Select RB-TnSeq mutants implied by our fitness data to be resistant to tailocins were isolated from their respective libraries and spotted with tailocin sample (see Methods). Target strains are: (A) Pse05 wild-type and mutants; (B) Pse03 wild-type and mutants; (C) Pse13 wild-type and mutants. An image of the presence or absence of a zone of clearance is shown. Some Pse13 mutants show differential sensitivity to tailocins from different producer strains. See Table S15 for more information on these mutants.

**
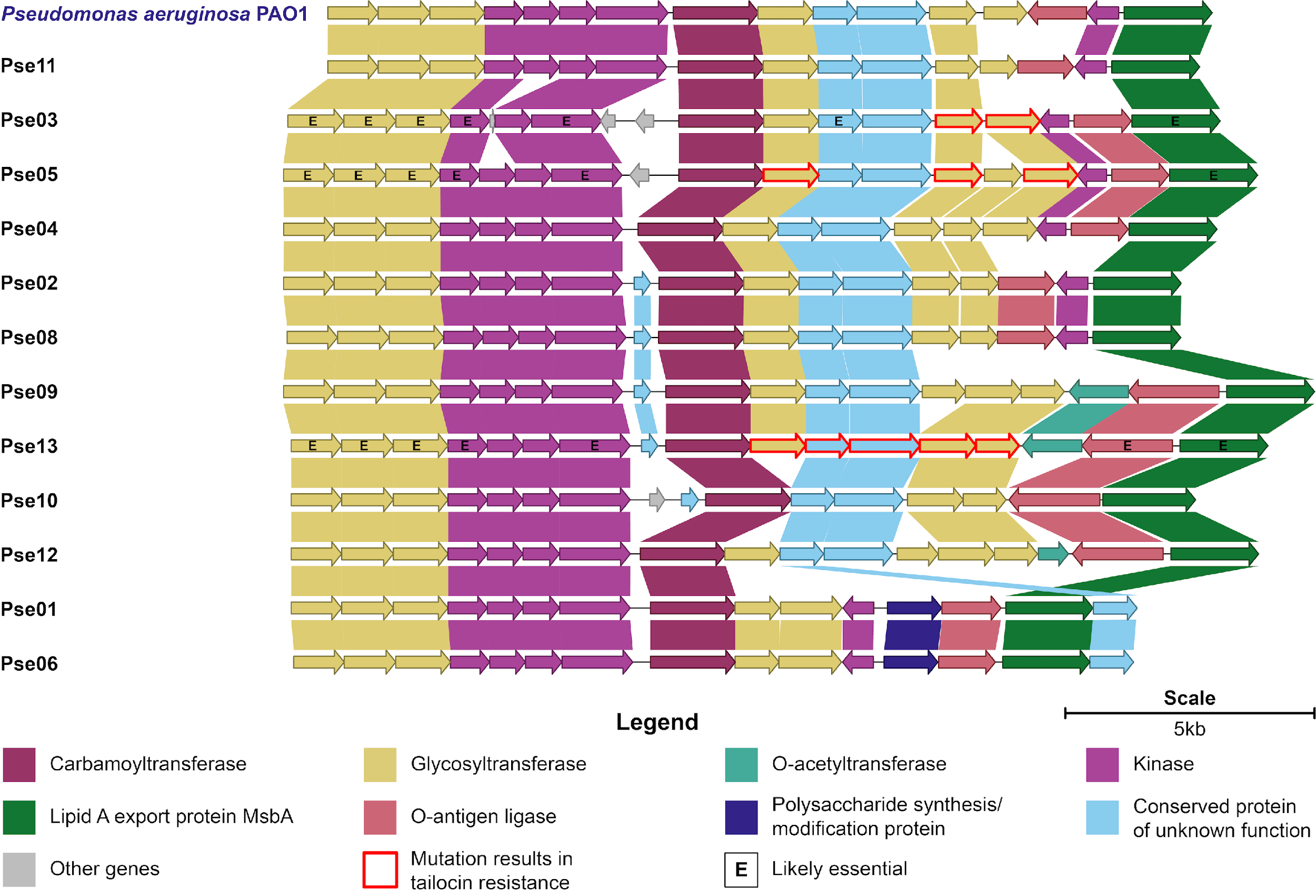
**

### Figure S5. LPS core oligosaccharide biosynthetic gene clusters. Illustrated and compared are the genes comprising LPS core clusters encoded by our 12 selected *Pseudomonas* isolates (black) and *P. aeruginosa* PAO1 (blue label). These clusters show a high degree of homology. Genes in the same orthogroup (Methods) are joined by a block of color. For a full list of orthogroups, see Table S16.

**
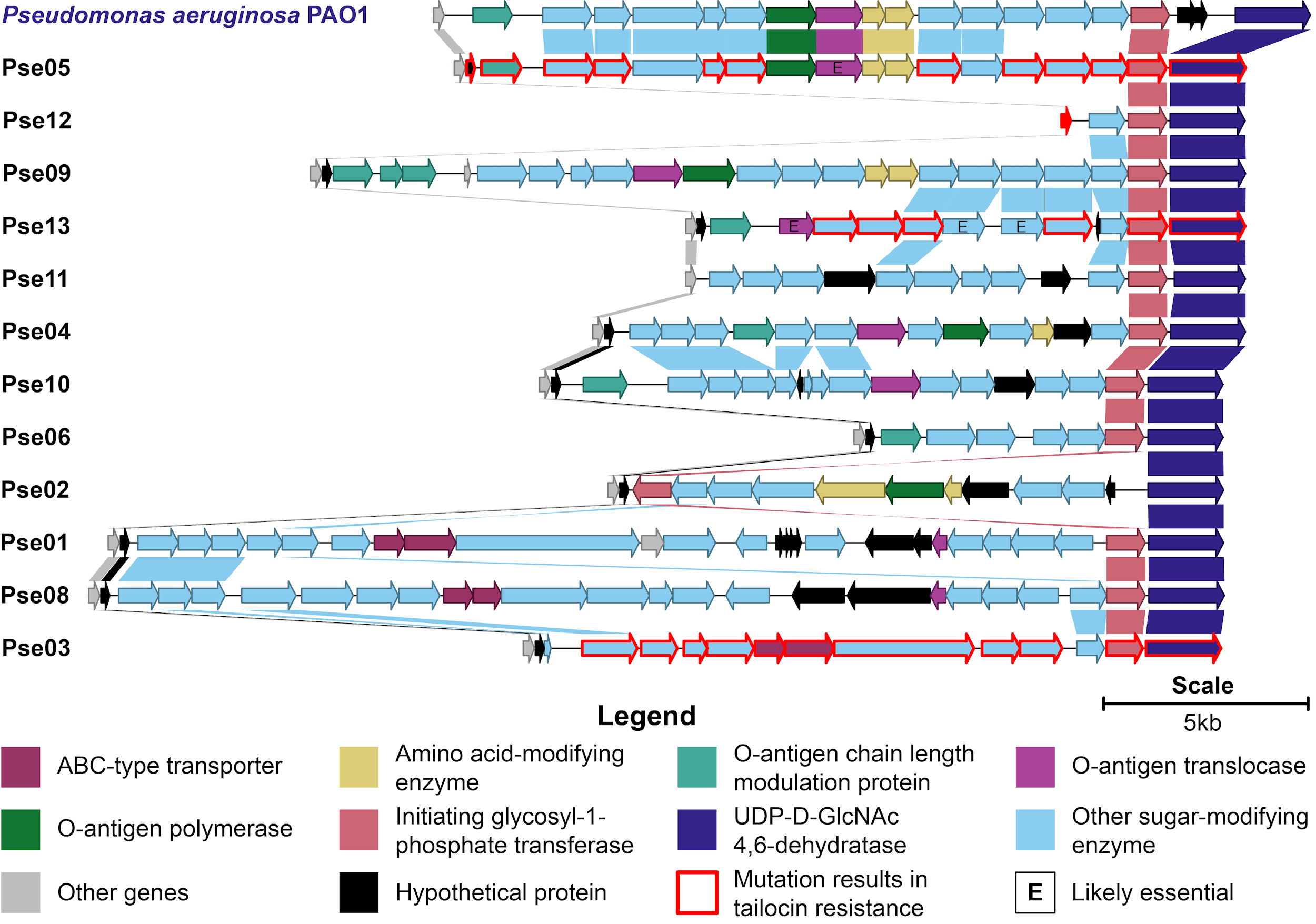
**

### Figure S6. O-specific antigen biosynthetic gene clusters. Illustrated and compared are the genes comprising OSA biosynthetic clusters encoded by our 12 selected *Pseudomonas* isolates (black) and *P. aeruginosa* PAO1 (blue label). While these clusters display considerable heterogeneity, do note the high homology between the clusters of PAO1 and Pse05. Genes in the same orthogroup (Methods) are joined by a block of color. For a full list of orthogroups, see Table S17.

**
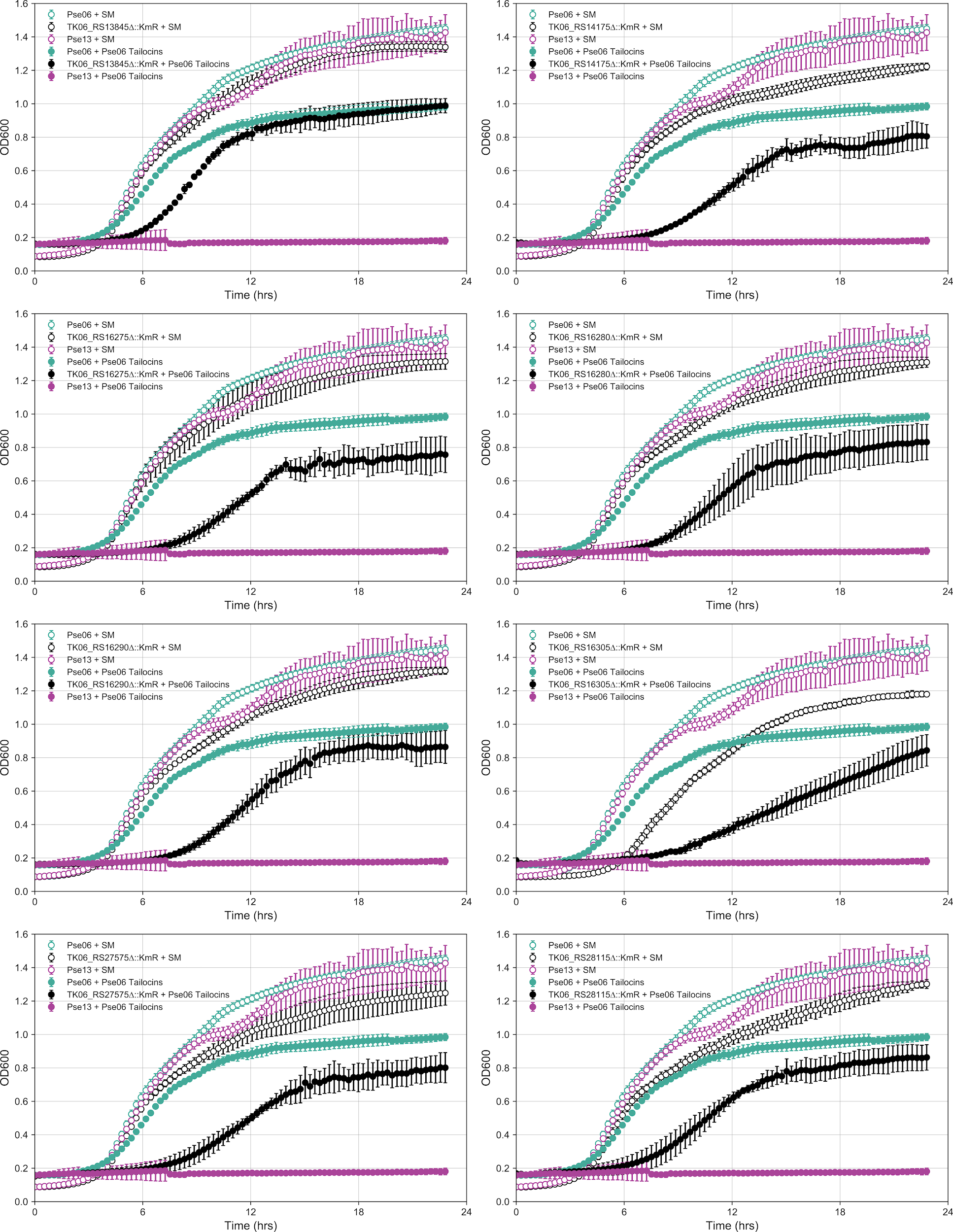
**

### Figure S7. Tailocin sensitivity phenotype validations. Mutations in Pse06 that cause increased tailocin sensitivity were regenerated via replacement of the gene with a kanamycin-resistance marker. Growth curves of these mutants, wild-type Pse06 (naturally resistant) and wild-type Pse13 (naturally sensitive) were obtained in the presence and absence of Pse06 tailocins. Growth assays proceeded for 22.5hrs. For each mutant, application of Pse06 tailocins results in weaker growth compared to wild-type Pse06, but does not eliminate growth like with wild-type Pse13. These experiments were repeated (triplicate) and averages are plotted. Error bars: standard deviation.

**
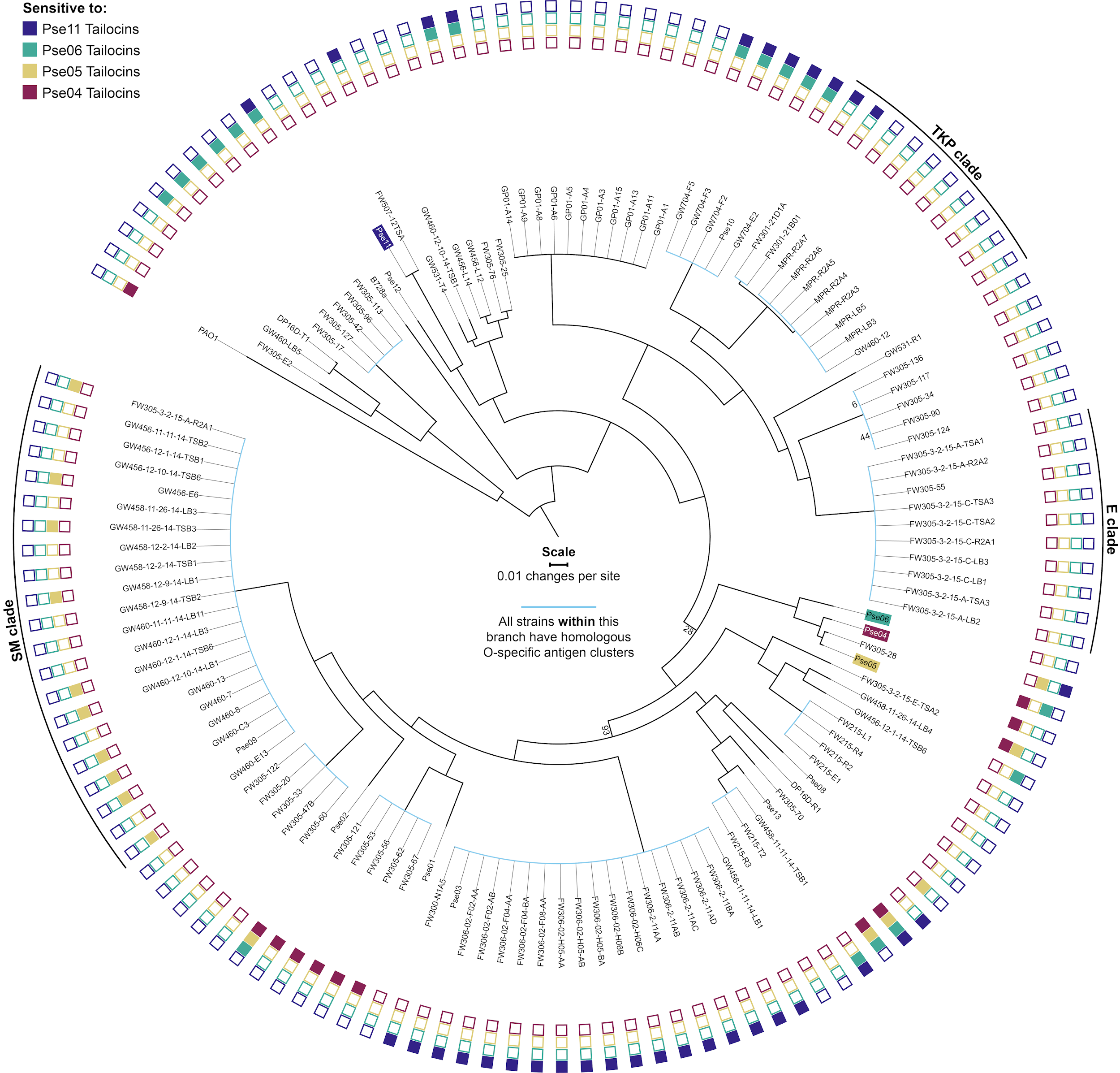
**

### Figure S8. Phylogenetic tree of target strains overlaid with tailocin sensitivity data. GTDB-Tk (Genome Taxonomy Database Toolkit)[^24^](https://paperpile.com/c/gPnyZb/ZWtHk) was used to find 88 ubiquitous single-copy genes found in each strain’s genome. Phylogenetic distances were then determined using a multilocus alignment of all 88 gene sequences (see Methods). The root of this tree is set to *P. aeruginosa* PAO1 in accordance with *Pseudomonas* phylogenomics[^67^](https://paperpile.com/c/gPnyZb/9O67U). All percentage bootstrap values <100% are labeled at branching nodes. Light blue colored branches indicate clades whose members all internally share a homologous O-specific antigen cluster, after we have annotated these clusters per Methods. Tailocin producing strain labels are highlighted in color. Shaded boxes at the outer edge of the tree indicate sensitivity of that strain to the correspondingly colored tailocin. To aid discussion of 3 clades of strains with interesting features, we named those clades after the most closely related species by 16S rRNA similarity: E clade (*P. extremorientalis*); TKP clade (*P.* sp. TKP); SM clade (*P. silesiensis*/*P.mandelii*). For the killing matrix in table format, see Table S21.

###

**
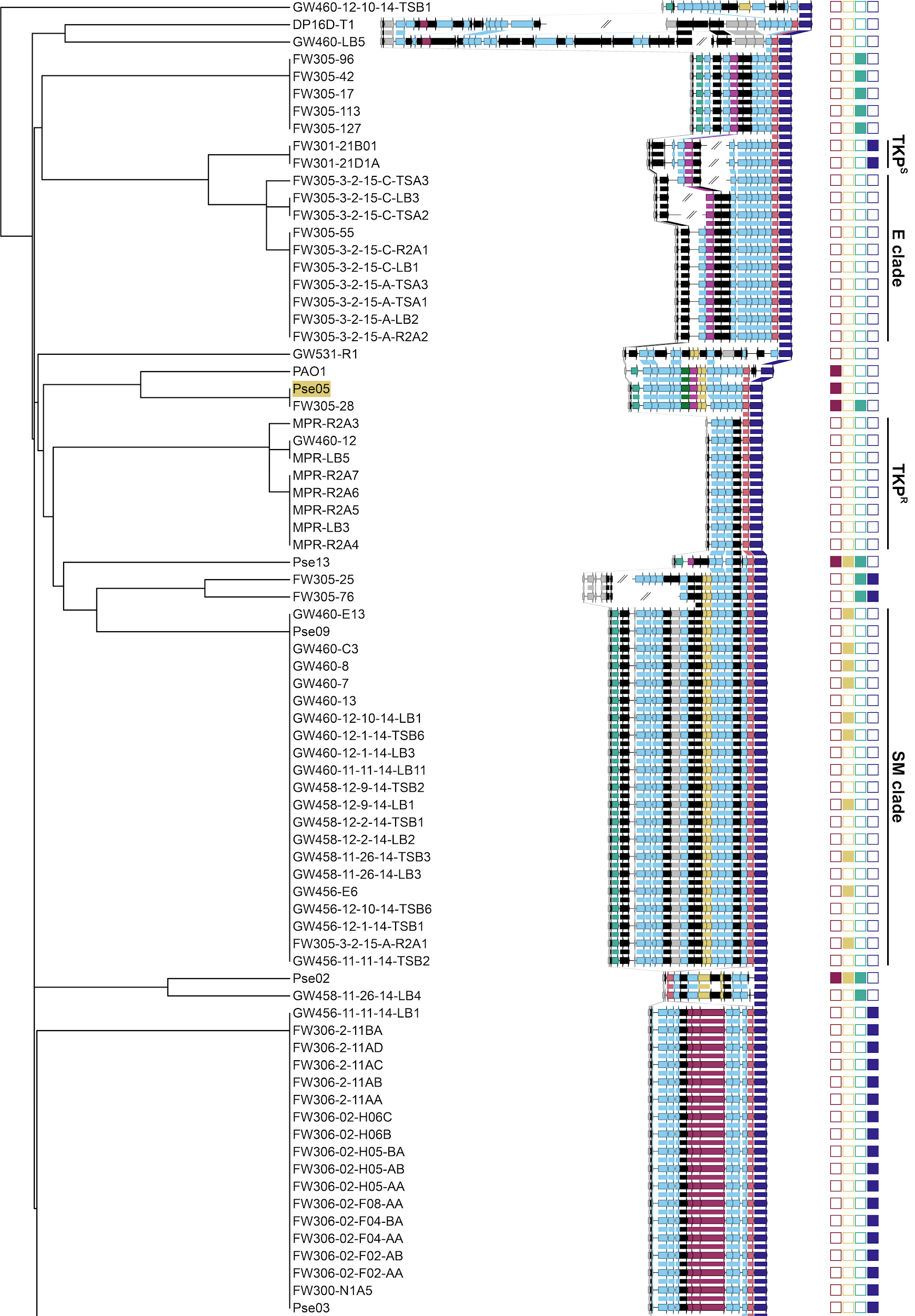
**

###
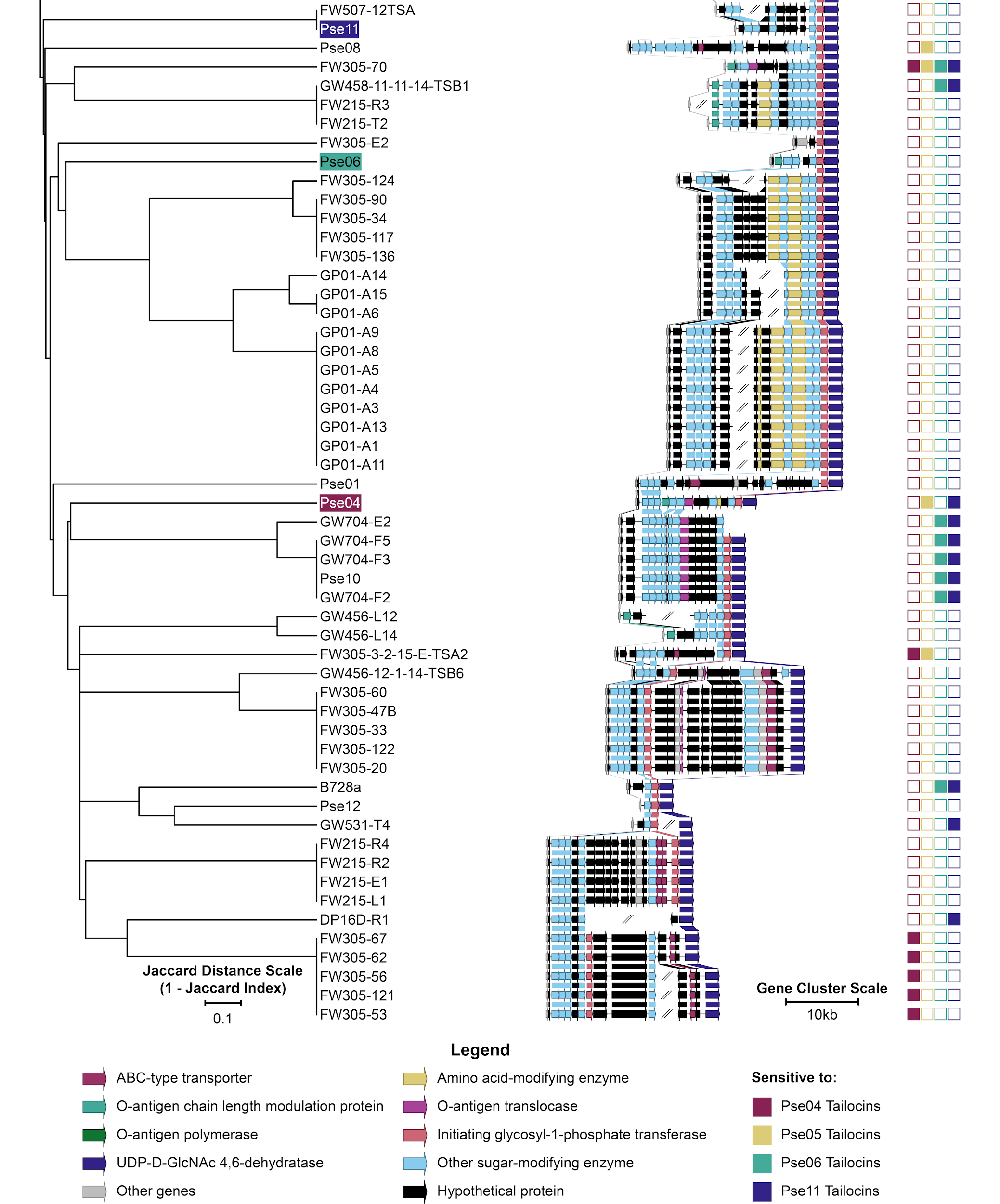


### Figure S9. O-specific antigen biosynthetic gene clusters of target strains overlaid with tailocin sensitivity data. Illustrations of the OSA clusters of all 130 *Pseudomonas* strains clustered in the same way as shown in Fig. 4. OSA clusters are defined as the region flanked by the genes *ihfB* (integration host factor subunit beta, left, non-inclusive) and *wbpM* (UDP-D-GlcNAc 4,6-dehydratase, right, inclusive). Genes in the same orthogroup (Methods) are joined by a block of color. Tailocin producing strain labels are highlighted in color. Shaded boxes on the right indicate sensitivity of that strain to the correspondingly colored tailocin. To aid discussion of 3 clades of strains with interesting features, we named those clades after the most closely related species by 16S rRNA similarity: E clade (*P. extremorientalis*); TKP clade (*P.* sp. TKP); SM clade (*P. silesiensis*/*P. mandelii*). In this clustering, TKP strains are separated into two groups: TKP^S^ (Pse11 Tailocins sensitive) and TKP^R^ (resistant). For the killing matrix in table format, see Table S21. For a full list of orthogroups, see Table S22.

## Supplementary Note 1. Review of the roles of LPS inner core biosynthetic enzymes on O-specific antigen display

Studies in *Escherichia coli* and *Salmonella enterica* show truncation of the LPS and elimination of OSA display following disruption of LPS inner core assembly genes (i.e. *waaA*, *waaC*, *waaF* and *waaG*)[^54^](https://paperpile.com/c/gPnyZb/lI0u1). However, we are unable to evaluate their effect on tailocin sensitivity in this study, since all of these genes are essential in *P. aeruginosa* PAO1[^34^](https://paperpile.com/c/gPnyZb/XDpfU) and likely essential (see Methods) in Pse05, Pse03 and Pse13. The one exception is *waaC* (AO353_RS12880) in Pse03, which confers neutral fitness to Pse11 tailocins when disrupted. This gene is disrupted just three times in the Pse03 RB-TnSeq library, with one transposon insertion at the 483rd base pair, and two insertions at the 531st base pair of this 1073bp gene. As the median number of insertions per gene in this library is eleven[^23^](https://paperpile.com/c/gPnyZb/O1oA), we postulate that the three non-lethal insertions in AO353_RS12880 may not have disrupted its essential LPS core assembly function, leaving O-specific antigen display unaffected. A viable transposon-insertion in *waaC* has been reported once before, in PAO1, in the 1038(1068)bp position, so this gene may tolerate select insertion sites[^64^](https://paperpile.com/c/gPnyZb/3YFdK).

## Supplementary Note 2. Putative functions of AO361_RS10865 and AO361_RS10900, genes involved in sensitivity to a subset of antagonistic tailocins

AO361_RS10865 encodes a putative glycosyltransferase but shares no significant similarity to any characterized enzyme. AO361_RS10900 is an ortholog of *wbpM* in PAO1 (78% identity, 100% coverage) and may share with *wbpM* a function in modifying OSA monomers. In PAO1, *wbpM* encodes a bifunctional, inner-membrane-bound sugar C6 dehydratase/C4 reductase that catalyzes the conversion of UDP-D-GlcNAc to UDP-4-keto-D-QuiNAc[^65^](https://paperpile.com/c/gPnyZb/CAEzM). This is an intermediary step in the biosynthesis of D-QuiNAc or D-FucNAc, monomers in the O-specific antigen (OSA) of *P. aeruginosa* serotypes O3, O5, O6 and O10. Deletion of *wbpM* in serotype O3, O5, O6 and O10 results in complete loss of the OSA. In contrast, deletion of *wbpM* in serotypes O15 and O17 results in slight modification to the OSA, as determined by silver-stained SDS-PAGE of LPS[^66^](https://paperpile.com/c/gPnyZb/d87nx). It is possible that AO361_RS10900 functions like *wbpM* in O15 and O17, performing a modification to the OSA of Pse13, and not assembling a core OSA structural component. That specific modification is likely a receptor of Pse05 and Pse04 tailocins, but not Pse06 tailocins.

# References

1. [Russel, J., Røder, H. L., Madsen, J. S., Burmølle, M. & Sørensen, S. J. Antagonism correlates with metabolic similarity in diverse bacteria. *Proc. Natl. Acad. Sci. U. S. A.* **114**, 10684–10688 (2017).](http://paperpile.com/b/gPnyZb/I1HBO)

2. [Riley, M. A. & Wertz, J. E. Bacteriocins: Evolution, Ecology, and Application. *Annu. Rev. Microbiol.* **56**, 117–137 (2002).](http://paperpile.com/b/gPnyZb/PEzBS)

3. [Scholl, D. Phage Tail–Like Bacteriocins. *Annual Review of Virology* **4**, 453–467 (2017).](http://paperpile.com/b/gPnyZb/2Cbdu)

4. [Nobrega, F. L. *et al.* Targeting mechanisms of tailed bacteriophages. *Nat. Rev. Microbiol.*](http://paperpile.com/b/gPnyZb/T0VKM) **16**, 760–773 [(2018) doi:](http://paperpile.com/b/gPnyZb/T0VKM)[10.1038/s41579-018-0070-8](http://dx.doi.org/10.1038/s41579-018-0070-8)[.](http://paperpile.com/b/gPnyZb/T0VKM)

5. [Baltrus, D. A., Clark, M., Smith, C. & Hockett, K. L. Localized recombination drives diversification of killing spectra for phage-derived syringacins. *ISME J.*](http://paperpile.com/b/gPnyZb/Lwx1P) **64**, 226–233 [(2018) doi:](http://paperpile.com/b/gPnyZb/Lwx1P)[10.1038/s41396-018-0261-3](http://dx.doi.org/10.1038/s41396-018-0261-3)[.](http://paperpile.com/b/gPnyZb/Lwx1P)

6. [Lee, G. *et al.* F-type bacteriocins of Listeria monocytogenes: A new class of phage tail-like structures reveals broad parallel coevolution between tailed bacteriophages and high-molecular-weight bacteriocins. *J. Bacteriol.* **198**, 2784–2793 (2016).](http://paperpile.com/b/gPnyZb/HdTM1)

7. [Scholl, D., Gebhart, D., Williams, S. R., Bates, A. & Mandrell, R. Genome sequence of E. coli O104:H4 leads to rapid development of a targeted antimicrobial agent against this emerging pathogen. *PLoS One* **7**, 1–5 (2012).](http://paperpile.com/b/gPnyZb/Eq2vT)

8. [Scholl, D. *et al.* An engineered R-type pyocin is a highly specific and sensitive bactericidal agent for the food-borne pathogen Escherichia coli O157:H7. *Antimicrob. Agents Chemother.* **53**, 3074–3080 (2009).](http://paperpile.com/b/gPnyZb/JDVOe)

9. [Williams, S. R., Gebhart, D., Martin, D. W. & Scholl, D. Retargeting R-type pyocins to generate novel bactericidal protein complexes. *Appl. Environ. Microbiol.* **74**, 3868–3876 (2008).](http://paperpile.com/b/gPnyZb/jae9Z)

10. [Ishii, S. I., Nishi, Y. & Egami, F. The fine structure of a pyocin. *J. Mol. Biol.* **13**, IN5–IN12 (1965).](http://paperpile.com/b/gPnyZb/5rfKK)

11. [Uratani, Y. & Hoshino, T. Pyocin R1 inhibits active transport in Pseudomonas aeruginosa and depolarizes membrane potential. *J. Bacteriol.* **157**, 632–636 (1984).](http://paperpile.com/b/gPnyZb/09JTH)

12. [Strauch, E. *et al.* Characterization of Enterocoliticin, a Phage Tail-Like Bacteriocin, and Its Effect on Pathogenic Yersinia enterocolitica Strains. *Appl. Environ. Microbiol.* **67**, 5634–5642 (2001).](http://paperpile.com/b/gPnyZb/gEUjE)

13. [Kohler, T., Donner, V. & van Delden, C. Lipopolysaccharide as Shield and Receptor for R-Pyocin-Mediated Killing in Pseudomonas aeruginosa. *J. Bacteriol.* **192**, 1921–1928 (2010).](http://paperpile.com/b/gPnyZb/jbpN4)

14. [Bashey, F., Young, S. K., Hawlena, H. & Lively, C. M. Spiteful interactions between sympatric natural isolates of Xenorhabdus bovienii benefit kin and reduce virulence. *J. Evol. Biol.* **25**, 431–437 (2012).](http://paperpile.com/b/gPnyZb/aCACz)

15. [Oluyombo, O., Penfold, C. N. & Diggle, S. P. Competition in Biofilms between Cystic Fibrosis Isolates of Pseudomonas aeruginosa Is Shaped by R-Pyocins. *MBio* **10**, e01828–18 (2019).](http://paperpile.com/b/gPnyZb/PYIIM)

16. [Ikeda, K. & Egami, F. Receptor Substance for Pyocin R. I. Partial Purification and Chemical Properties. *J. Biochem.* **65**, 603–609 (1969).](http://paperpile.com/b/gPnyZb/6n0Iq)

17. [Govan, J. R. W. Studies on the Pyocins of Pseudomonas aeruginosa: Morphology and Mode of Action of Contractile Pyocins. *J. Gen. Microbiol.* **80**, 1–15 (1974).](http://paperpile.com/b/gPnyZb/80hFF)

18. [Meadow, P. M. & Wells, P. L. Receptor Sites for R-type Pyocins and Bacteriophage E79 in the Core Part of the Lipopolysaccharide of Pseudomonas aeruginosa PAC1. *J. Gen. Microbiol.* **108**, 339–343 (1978).](http://paperpile.com/b/gPnyZb/0Yn91)

19. [Ritchie, J. M. *et al.* An Escherichia coli O157-specific engineered pyocin prevents and ameliorates infection by E. coli O157:H7 in an animal model of diarrheal disease. *Antimicrob. Agents Chemother.* **55**, 5469–5474 (2011).](http://paperpile.com/b/gPnyZb/fH7Uo)

20. [Filiatrault, M. J., Munson R.S., J. & Campagnari, A. A. Genetic analysis of a pyocin-resistant lipooligosaccharide (LOS) mutant of Haemophilus ducreyi: Restoration of full-length LOS restores pyocin sensitivity. *J. Bacteriol.* **183**, 5756–5761 (2001).](http://paperpile.com/b/gPnyZb/PmXWq)

21. [Hockett, K. L., Clark, M., Scott, S. & Baltrus, D. A. Conditionally Redundant Bacteriocin Targeting by Pseudomonas syringae. *bioRxiv* (2017) doi:](http://paperpile.com/b/gPnyZb/OqzoY)[10.1101/167593](http://dx.doi.org/10.1101/167593)[.](http://paperpile.com/b/gPnyZb/OqzoY)

22. [Wetmore, K. M. *et al.* Rapid Quantification of Mutant Fitness in Diverse Bacteria by Sequencing Randomly Bar-Coded Transposons. *MBio* **6**, e00306–15 (2015).](http://paperpile.com/b/gPnyZb/g2xkC)

23. [Price, M. N. *et al.* Mutant phenotypes for thousands of bacterial genes of unknown function. *Nature* 1 (2018) doi:](http://paperpile.com/b/gPnyZb/O1oA)[10.1038/s41586-018-0124-0](http://dx.doi.org/10.1038/s41586-018-0124-0)[.](http://paperpile.com/b/gPnyZb/O1oA)

24. [Chaumeil, P.-A., Mussig, A. J., Hugenholtz, P. & Parks, D. H. GTDB-Tk: a toolkit to classify genomes with the Genome Taxonomy Database. *Bioinformatics* **36**, 1925–1927 (2019).](http://paperpile.com/b/gPnyZb/ZWtHk)

25. [Loper, J. E. *et al.* Comparative genomics of plant-associated pseudomonas spp.: Insights into diversity and inheritance of traits involved in multitrophic interactions. *PLoS Genet.* **8**, e1002784 (2012).](http://paperpile.com/b/gPnyZb/W6usR)

26. [Hockett, K. L., Renner, T. & Baltrus, D. A. Independent co-option of a tailed bacteriophage into a killing complex in Pseudomonas. *MBio* **6**, 1–11 (2015).](http://paperpile.com/b/gPnyZb/NvzKz)

27. [Ghequire, M. G. K. *et al.* Different Ancestries of R Tailocins in Rhizospheric Pseudomonas Isolates. *Genome Biol. Evol.* **7**, 2810–2828 (2015).](http://paperpile.com/b/gPnyZb/Vq7di)

28. [Nakayama, K. *et al.* The R-type pyocin of Pseudomonas aeruginosa is related to P2 phage, and the F-type is related to lambda phage. *Mol. Microbiol.* **38**, 213–231 (2000).](http://paperpile.com/b/gPnyZb/dmnRV)

29. [Mavrodi, D. V., Loper, J. E., Paulsen, I. T. & Thomashow, L. S. Mobile genetic elements in the genome of the beneficial rhizobacterium Pseudomonas fluorescens Pf-5. *BMC Microbiol.* **9**, 1–18 (2009).](http://paperpile.com/b/gPnyZb/uVIMz)

30. [Arndt, D. *et al.* PHASTER: a better, faster version of the PHAST phage search tool. *Nucleic Acids Res.* **44**, W16–W21 (2016).](http://paperpile.com/b/gPnyZb/UDeWg)

31. [Higerd, T. B., Baechler, C. A. & Berk, R. S. Morphological studies on relaxed and contracted forms of purified pyocin particles. *J. Bacteriol.* **98**, 1378–1389 (1969).](http://paperpile.com/b/gPnyZb/qQnBa)

32. [Dorosky, R. J., Yu, J. M., Pierson, L. S. & Pierson, E. A. Pseudomonas chlororaphis Produces Two Distinct R-Tailocins That Contribute to Bacterial Competition in Biofilms and on Roots. *Appl. Environ. Microbiol.* **83**, 1–16 (2017).](http://paperpile.com/b/gPnyZb/ezLx2)

33. [Dorosky, R. J., Pierson, L. S. & Pierson, E. A. *Pseudomonas chlororaphis* produces multiple R-tailocin particles that broaden the killing spectrum and contribute to persistence in rhizosphere communities. *Appl. Environ. Microbiol.* **84**, AEM.01230–18 (2018).](http://paperpile.com/b/gPnyZb/n1qPl)

34. [King, J. D., Kocíncová, D., Westman, E. L. & Lam, J. S. Review: lipopolysaccharide biosynthesis in Pseudomonas aeruginosa. *Innate Immun.* **15**, 261–312 (2009).](http://paperpile.com/b/gPnyZb/XDpfU)

35. [Mutalik, V. K. *et al.* High-throughput mapping of the phage resistance landscape in E. coli. *bioRxiv*](http://paperpile.com/b/gPnyZb/hw4Xm) 2020.02.15.951020 [(2020).](http://paperpile.com/b/gPnyZb/hw4Xm)

36. [Adler, B. A. *et al.* Systematic Discovery of Salmonella Phage-Host Interactions via High-Throughput Genome-Wide Screens. *bioRxiv* 2020.04.27.058388 (2020) doi:](http://paperpile.com/b/gPnyZb/lZicm)[10.1101/2020.04.27.058388](http://dx.doi.org/10.1101/2020.04.27.058388)[.](http://paperpile.com/b/gPnyZb/lZicm)

37. [Kandel, P. P., Baltrus, D. A. & Hockett, K. L. Pseudomonas Can Survive Tailocin Killing via Persistence-like and Heterogenous Resistance Mechanisms. *J. Bacteriol.* (2020) doi:](http://paperpile.com/b/gPnyZb/4Z4g4)[10.1128/JB.00142-20](http://dx.doi.org/10.1128/JB.00142-20)[.](http://paperpile.com/b/gPnyZb/4Z4g4)

38. [Kocíncová, D. & Lam, J. S. A deletion in the wapB promoter in many serotypes of Pseudomonas aeruginosa accounts for the lack of a terminal glucose residue in the core oligosaccharide and resistance to killing by R3-pyocin. *Mol. Microbiol.* **89**, 464–478 (2013).](http://paperpile.com/b/gPnyZb/ZgPJS)

39. [Bertozzi Silva, J., Storms, Z. & Sauvageau, D. Host receptors for bacteriophage adsorption. *FEMS Microbiol. Lett.* **363**, 1–11 (2016).](http://paperpile.com/b/gPnyZb/lFpvu)

40. [Urtecho, G., Campbell, D. E., Hershey, D. M., Whitaker, R. J. & O’Toole, G. A. Discovering the Molecular Determinants of Phaeobacter inhibens susceptibility to Phaeobacter phage MD18. *bioRxiv* (2020).](http://paperpile.com/b/gPnyZb/lyyQB)

41. [Ekiert, D. C. *et al.* Architectures of Lipid Transport Systems for the Bacterial Outer Membrane. *Cell* **169**, 273–285.e17 (2017).](http://paperpile.com/b/gPnyZb/9NLqF)

42. [Powers, M. J. & Trent, M. S. Phospholipid retention in the absence of asymmetry strengthens the outer membrane permeability barrier to last-resort antibiotics. *Proc. Natl. Acad. Sci. U. S. A.* **115**, E8518–E8527 (2018).](http://paperpile.com/b/gPnyZb/lMVLI)

43. [Shrivastava, R. & Chng, S.-S. Lipid trafficking across the Gram-negative cell envelope. *J. Biol. Chem.* **294**, 14175–14184 (2019).](http://paperpile.com/b/gPnyZb/qAujy)

44. [Sperandeo, P., Martorana, A. M. & Polissi, A. The lipopolysaccharide transport (Lpt) machinery: A nonconventional transporter for lipopolysaccharide assembly at the outer membrane of Gram-negative bacteria. *J. Biol. Chem.* **292**, 17981–17990 (2017).](http://paperpile.com/b/gPnyZb/moHXD)

45. [Narita, S.-I., Masui, C., Suzuki, T., Dohmae, N. & Akiyama, Y. Protease homolog BepA (YfgC) promotes assembly and degradation of β-barrel membrane proteins in Escherichia coli. *Proc. Natl. Acad. Sci. U. S. A.* **110**, E3612–21 (2013).](http://paperpile.com/b/gPnyZb/mQ6cP)

46. [Teran, W. *et al.* Antibiotic-Dependent Induction of Pseudomonas putida DOT-T1E TtgABC Efflux Pump Is Mediated by the Drug Binding Repressor TtgR. *Antimicrob. Agents Chemother.* **47**, 3067–3072 (2003).](http://paperpile.com/b/gPnyZb/GxsoH)

47. [Liang, H., Li, L., Dong, Z., Surette, M. G. & Duan, K. The YebC family protein PA0964 negatively regulates the Pseudomonas aeruginosa quinolone signal system and pyocyanin production. *J. Bacteriol.* **190**, 6217–6227 (2008).](http://paperpile.com/b/gPnyZb/r4f3h)

48. [Ghequire, M. G. K., Kemland, L. & De Mot, R. Novel Immunity Proteins Associated with Colicin M-like Bacteriocins Exhibit Promiscuous Protection in Pseudomonas. *Front. Microbiol.* **8**, 1–9 (2017).](http://paperpile.com/b/gPnyZb/2anB9)

49. [Alvarez-Sieiro, P., Montalbán-López, M., Mu, D. & Kuipers, O. P. Bacteriocins of lactic acid bacteria: extending the family. *Appl. Microbiol. Biotechnol.* **100**, 2939–2951 (2016).](http://paperpile.com/b/gPnyZb/gZ890)

50. [Sgro, G. G. *et al.* Bacteria-Killing Type IV Secretion Systems. *Front. Microbiol.* **10**, 1–20 (2019).](http://paperpile.com/b/gPnyZb/mc9Ou)

51. [Yang, X., Long, M. & Shen, X. Effector–Immunity Pairs Provide the T6SS Nanomachine its Offensive and Defensive Capabilities. *Molecules* **23**, 1009 (2018).](http://paperpile.com/b/gPnyZb/fziPJ)

52. [Emms, D. M. & Kelly, S. OrthoFinder: phylogenetic orthology inference for comparative genomics. *Genome Biology* **20**, 238 (2019).](http://paperpile.com/b/gPnyZb/oqzaS)

53. [Jaillard, M. *et al.* A fast and agnostic method for bacterial genome-wide association studies: Bridging the gap between k-mers and genetic events. *PLoS Genet.* **14**, 1–28 (2018).](http://paperpile.com/b/gPnyZb/B9lxn)

54. [Bertani, B. & Ruiz, N. Function and Biogenesis of Lipopolysaccharides. *EcoSal Plus* **8**, 1–19 (2018).](http://paperpile.com/b/gPnyZb/lI0u1)

55. [Broeker, N. K. & Barbirz, S. Not a barrier but a key: How bacteriophages exploit host’s O-antigen as an essential receptor to initiate infection. *Mol. Microbiol.* **105**, 353–357 (2017).](http://paperpile.com/b/gPnyZb/TWvrz)

56. [Mutalik, V. K. *et al.* Dual-barcoded shotgun expression library sequencing for high-throughput characterization of functional traits in bacteria. *Nat. Commun.* **10**, 1–14 (2019).](http://paperpile.com/b/gPnyZb/9e1T5)

57. [Rishi, H. S. *et al.* Systematic genome-wide querying of coding and non-coding functional elements in E. coli using CRISPRi. *bioRxiv* (2020) doi:](http://paperpile.com/b/gPnyZb/kNqvK)[10.1101/2020.03.04.975888](http://dx.doi.org/10.1101/2020.03.04.975888)[.](http://paperpile.com/b/gPnyZb/kNqvK)

58. [High, N. J., Deadman, M. E. & Moxon, E. R. The role of a repetitive DNA motif (5’-CAAT-3') in the variable expression of the Haemophilus influenzae lipopolysaccharide epitope αGal(1–4)βGal. *Mol. Microbiol.* **9**, 1275–1282 (1993).](http://paperpile.com/b/gPnyZb/PpCPr)

59. [Broadbent, S. E., Davies, M. R. & Van Der Woude, M. W. Phase variation controls expression of Salmonella lipopolysaccharide modification genes by a DNA methylation-dependent mechanism. *Mol. Microbiol.* **77**, 337–353 (2010).](http://paperpile.com/b/gPnyZb/RKMgP)

60. [Kim, M. & Ryu, S. Spontaneous and transient defence against bacteriophage by phase-variable glucosylation of O-antigen in Salmonella enterica serovar Typhimurium. *Mol. Microbiol.* **86**, 411–425 (2012).](http://paperpile.com/b/gPnyZb/ts1Jz)

61. [Seed, K. D. *et al.* Phase Variable O Antigen Biosynthetic Genes Control Expression of the Major Protective Antigen and Bacteriophage Receptor in Vibrio cholerae O1. *PLoS Pathog.* **8**, e1002917 (2012).](http://paperpile.com/b/gPnyZb/nuLuv)

62. [Cota, I. *et al.* Epigenetic Control of Salmonella enterica O-Antigen Chain Length: A Tradeoff between Virulence and Bacteriophage Resistance. *PLoS Genet.* **11**, e1005667 (2015).](http://paperpile.com/b/gPnyZb/BmXl1)

63. [Turkington, C. J. R., Morozov, A., Clokie, M. R. J. & Bayliss, C. D. Phage-resistant phase-variant sub-populations mediate herd immunity against bacteriophage invasion of bacterial meta-populations. *Front. Microbiol.* **10**, 1–14 (2019).](http://paperpile.com/b/gPnyZb/yWrE4)

64. [Jacobs, M. A. *et al.* Comprehensive transposon mutant library of Pseudomonas aeruginosa. *Proceedings of the National Academy of Sciences* **100**, 14339–14344 (2003).](http://paperpile.com/b/gPnyZb/3YFdK)

65. [Creuzenet, C. & Lam, J. S. Topological and functional characterization of WbpM, an inner membrane UDP-GlcNAc C6 dehydratase essential for lipopolysaccharide biosynthesis in Pseudomonas aeruginosa. *Mol. Microbiol.* **41**, 1295–1310 (2001).](http://paperpile.com/b/gPnyZb/CAEzM)

66. [Burrows, L. L., Urbanic, R. V. & Lam, J. S. Functional conservation of the polysaccharide biosynthetic protein WbpM and its homologues in Pseudomonas aeruginosa and other medically significant bacteria. *Infect. Immun.* **68**, 931–936 (2000).](http://paperpile.com/b/gPnyZb/d87nx)

67. [Gomila, M., Peña, A., Mulet, M., Lalucat, J. & Garcí­a-Valdés, E. Phylogenomics and systematics in Pseudomonas. *Front. Microbiol.* **6**, 519–525 (2015).](http://paperpile.com/b/gPnyZb/9O67U)

68. [Lennox, E. S. Transduction of linked genetic characters of the host by bacteriophage P1. *Virology* **1**, 190–206 (1955).](http://paperpile.com/b/gPnyZb/6m6qO)

69. [Holloway, B. W. Genetic Recombination in Pseudomonas aeruginosa. *Microbiology* **13**, 572–581 (1955).](http://paperpile.com/b/gPnyZb/WoVE)

70. [Loper, J. E. & Lindow, S. E. Lack of Evidence for In Situ Fluorescent Pigment Production by Pseudomonas syringae pv. syringae on Bean Leaf Surfaces. *Phytopathology* **77**, 1449-1454 (1987).](http://paperpile.com/b/gPnyZb/ajhY)

71. [Thorgersen, M. P. *et al.* Molybdenum availability is key to nitrate removal in contaminated groundwater environments. *Appl. Environ. Microbiol.* **81**, 4976–4983 (2015).](http://paperpile.com/b/gPnyZb/K6X5)

72. [Carlson, H. K. *et al.* The selective pressures on the microbial community in a metal-contaminated aquifer. *ISME J.* **13**, 937–949 (2019).](http://paperpile.com/b/gPnyZb/nZZ1)

73. [Bankevich, A. *et al.* SPAdes: A New Genome Assembly Algorithm and Its Applications to Single-Cell Sequencing. *J. Comput. Biol.* **19**, 455–477 (2012).](http://paperpile.com/b/gPnyZb/6XStA)

74. [Guy, L., Kultima, J. R., Andersson, S. G. E. & Quackenbush, J. GenoPlotR: comparative gene and genome visualization in R. *Bioinformatics* **27**, 2334–2335 (2011).](http://paperpile.com/b/gPnyZb/r0uE6)

75. [Washburn, M. P., Wolters, D. & Yates, J. R., 3rd. Large-scale analysis of the yeast proteome by multidimensional protein identification technology. *Nat. Biotechnol.* **19**, 242–247 (2001).](http://paperpile.com/b/gPnyZb/UPNJI)

76. [Ko, H., Soo, K., Candlin, J. & Chiang, D. A New Method of Generating Decoy Peptides in SEQUEST to Quantify False-Positive Rates for Peptide Results. in *HUPO 5th Annual World Congress* 669 (2006).](http://paperpile.com/b/gPnyZb/LNQ1a)

77. [Cociorva, D., Tabb, D. L. & Yates, J. R. Validation of Tandem Mass Spectrometry Database Search Results Using DTASelect. *Curr. Protoc. Bioinformatics* **16**, 1–14 (2006).](http://paperpile.com/b/gPnyZb/GMxuP)

78. [Tabb, D. L., McDonald, W. H. & Yates, J. R. DTASelect and contrast: Tools for assembling and comparing protein identifications from shotgun proteomics. *J. Proteome Res.* **1**, 21–26 (2002).](http://paperpile.com/b/gPnyZb/HsV1l)

79. [Park, S. K., Venable, J. D., Xu, T. & Yates, J. R. A quantitative analysis software tool for mass spectrometry-based proteomics. *Nat. Methods* **5**, 319–322 (2008).](http://paperpile.com/b/gPnyZb/GnCa2)

80. [McDonald, W. H. *et al.* MS1, MS2, and SQT - Three unified, compact, and easily parsed file formats for the storage of shotgun proteomic spectra and identifications. *Rapid Commun. Mass Spectrom.* **18**, 2162–2168 (2004).](http://paperpile.com/b/gPnyZb/Qri6y)

81. [Peng, J., Elias, J. E., Thoreen, C. C., Licklider, L. J. & Gygi, S. P. Evaluation of multidimensional chromatography coupled with tandem mass spectrometry (LC/LC-MS/MS) for large-scale protein analysis: The yeast proteome. *J. Proteome Res.* **2**, 43–50 (2003).](http://paperpile.com/b/gPnyZb/nN2Eg)

82. [Ishihama, Y. *et al.* Exponentially Modified Protein Abundance Index (emPAI) for Estimation of Absolute Protein Amount in Proteomics by the Number of Sequenced Peptides per Protein. *Mol. Cell. Proteomics* **4**, 1265–1272 (2005).](http://paperpile.com/b/gPnyZb/PdQQc)

83. [Choi, K. H., Kumar, A. & Schweizer, H. P. A 10-min method for preparation of highly electrocompetent Pseudomonas aeruginosa cells: Application for DNA fragment transfer between chromosomes and plasmid transformation. *J. Microbiol. Methods* **64**, 391–397 (2006).](http://paperpile.com/b/gPnyZb/rhZnO)

84. [Liu, Z. *et al.* A Genome-Wide Screen Identifies Genes in Rhizosphere-Associated Pseudomonas Required to Evade Plant Defenses. *MBio* **9**, e00433-18 (2018).](http://paperpile.com/b/gPnyZb/cwinU)

85. [Lam, J. S., Taylor, V. L., Islam, S. T., Hao, Y. & Kocíncová, D. Genetic and functional diversity of Pseudomonas aeruginosa lipopolysaccharide. *Front. Microbiol.* **2**, 1–25 (2011).](http://paperpile.com/b/gPnyZb/ENy7y)

86. [Letunic, I. & Bork, P. Interactive Tree Of Life (iTOL) v4: recent updates and new developments. *Nucleic Acids Res.* **47**, W256–W259 (2019).](http://paperpile.com/b/gPnyZb/QodNu)

87. [Katoh, K. & Standley, D. M. MAFFT multiple sequence alignment software version 7: Improvements in performance and usability. *Mol. Biol. Evol.* **30**, 772–780 (2013).](http://paperpile.com/b/gPnyZb/TqIKR)

88. [Price, M. N., Dehal, P. S. & Arkin, A. P. FastTree 2 - Approximately maximum-likelihood trees for large alignments. *PLoS One* **5**, e9490 (2010).](http://paperpile.com/b/gPnyZb/U40wl)
